# Comparative prognostic accuracy of sepsis scores for hospital mortality in adults with suspected infection in non-ICU and ICU at an academic public hospital

**DOI:** 10.1371/journal.pone.0222563

**Published:** 2019-09-16

**Authors:** Christopher P. Kovach, Grant S. Fletcher, Kristina E. Rudd, Rosemary M. Grant, David J. Carlbom

**Affiliations:** 1 Division of Pulmonary, Critical Care, and Sleep Medicine, Department of Medicine, University of Washington, Seattle, Washington, United States of America; 2 Division of Hospital Medicine, Department of Medicine, University of Washington, Seattle, Washington, United States of America; 3 Clinical Research, Investigation, and Systems Modeling of Acute Illness (CRISMA) Center, Department of Critical Care Medicine, University of Pittsburgh, Pittsburgh, Pennsylvania, United States of America; 4 Professional Development and Nursing Excellence, Harborview Medical Center, Seattle, Washington, United States of America; Fundacao Oswaldo Cruz, BRAZIL

## Abstract

**Background:**

Sepsis is a global healthcare challenge and reliable tools are needed to identify patients and stratify their risk. Here we compare the prognostic accuracy of the sepsis-related organ failure assessment (SOFA), quick SOFA (qSOFA), systemic inflammatory response syndrome (SIRS), and national early warning system (NEWS) scores for hospital mortality and other outcomes amongst patients with suspected infection at an academic public hospital.

**Measurements and main results:**

10,981 adult patients with suspected infection hospitalized at a U.S. academic public hospital between 2011–2017 were retrospectively identified. Primary exposures were the maximum SIRS, qSOFA, SOFA, and NEWS scores upon inclusion. Comparative prognostic accuracy for the primary outcome of hospital mortality was assessed using the area under the receiver operating characteristic curve (AUROC). Secondary outcomes included mortality in ICU versus non-ICU settings, ICU transfer, ICU length of stay (LOS) >3 days, and hospital LOS >7 days. Adjusted analyses were performed using a model of baseline risk for hospital mortality. 774 patients (7.1%) died in hospital. Discrimination for hospital mortality was highest for SOFA (AUROC 0.90 [95% CI, 0.89–0.91]), followed by NEWS (AUROC 0.85 [95% CI, 0.84–0.86]), qSOFA (AUROC 0.84 [95% CI, 0.83–0.85]), and SIRS (AUROC 0.79 [95% CI, 0.78–0.81]; p<0.001 for all comparisons). NEWS (AUROC 0.94 [95% CI, 0.93–0.95]) outperformed other scores in predicting ICU transfer (qSOFA AUROC 0.89 [95% CI, 0.87–0.91]; SOFA AUROC, 0.84 [95% CI, 0.82–0.87]; SIRS AUROC 0.81 [95% CI, 0.79–0.83]; p<0.001 for all comparisons). NEWS (AUROC 0.86 [95% CI, 0.85–0.86]) was also superior to other scores in predicting ICU LOS >3 days (SOFA AUROC 0.84 [95% CI, 0.83–0.85; qSOFA AUROC, 0.83 [95% CI, 0.83–0.84]; SIRS AUROC, 0.75 [95% CI, 0.74–0.76]; p<0.002 for all comparisons).

**Conclusions:**

Multivariate prediction scores, such as SOFA and NEWS, had greater prognostic accuracy than qSOFA or SIRS for hospital mortality, ICU transfer, and ICU length of stay. Complex sepsis scores may offer enhanced prognostic performance as compared to simple sepsis scores in inpatient hospital settings where more complex scores can be readily calculated.

## Introduction

Sepsis is a major healthcare challenge in the United States and globally, and is associated with profound mortality, morbidity, and healthcare costs [1–7]. Early recognition and treatment of sepsis improves outcomes; reliable tools are needed to identify patients at increased risk of developing sepsis and to prognosticate their mortality and other complications [8, 9]. Sepsis-3 authors recommend the quick sepsis-related organ failure assessment (qSOFA) score to identify patients at high risk of developing sepsis outside the intensive care unit (ICU) and the SOFA score for patients in the ICU [10, 11]. Internal and external validation studies have demonstrated the superiority of the qSOFA and SOFA scores for the identification and mortality prognostication of sepsis patients, when compared to the systemic inflammatory response syndrome (SIRS) criteria [10, 12–15]. While the Sepsis-3 authors initially proposed the qSOFA and SOFA scores as tools to identify patients with organ dysfunction among those with suspected infection, there is widespread interest in using these and other scores in prognosticating patient outcomes secondary to sepsis [16–21]. A recent meta-analysis comparing the qSOFA score with SIRS criteria concluded that the qSOFA score was more predictive of hospital mortality but SIRS was superior for sepsis diagnosis [22]. However, other studies have shown that alternative scores, such as the national early warning score (NEWS) may be superior [20, 23–26]. The ideal sepsis identification and outcome prognostication scoring system remains uncertain. We compared the prognostic accuracy of sepsis scores for hospital mortality among patients with suspected infection presenting to the emergency department (ED) and then admitted to either the acute care service or ICU of an academic public hospital. We hypothesized that there were important differences between scores which may impact score performance among different hospitalized populations.

## Methods

### Study design and population

A retrospective cohort study was performed using all patients ≥18 years of age with suspected infection who presented to the ED and were admitted to Harborview Medical Center, a tertiary academic public hospital in Seattle, WA with 413 beds, between January 2011 and March 2017. Patients were identified on the basis of suspected infection given that there is no gold standard for the diagnosis of sepsis [10]. Suspected infection was defined as (1) any blood, urine, or sputum culture order followed by clinician order of an intravenous (IV) antibiotic within 72 hours, or (2) clinician order of an IV antibiotic followed by a culture order within 24 hours. This method was chosen for consistency with recent major sepsis studies [7, 10, 14, 15, 23]. All patients in the ED, acute care service, or ICU were eligible for inclusion in the study. The time at which a patient met the definition of suspected infection was used as the time of study inclusion. Primary exposures were the maximum SIRS, qSOFA, SOFA, and NEWS scores upon inclusion. Patients who were directly admitted to the hospital without being evaluated in the ED, those transferred from the ED or inpatient wards of another hospital, those who were evaluated in the ED and then discharged, and those admitted to inpatient psychiatric or rehabilitation services were excluded. The study was approved by the University of Washington Institutional Review Board (IRB #00002870).

### Data collection

Patient demographic data, vital signs, laboratory values, orders (e.g., medications, cultures, oxygen therapy, vasopressors), hospital mortality data, and ICU and hospital length of stay (LOS) were extracted from the electronic health record, de-identified, and made available on a secure server for analysis. Each patient’s qSOFA, SOFA, SIRS, and NEWS scores were calculated at time of inclusion in the study using the most deranged physiologic and laboratory parameters recorded within the 24 hours preceding and the 24 hours following time of inclusion [11, 12, 25, 27]. Standard criteria for score positivity were applied, using a threshold of 2 or more points for each of SIRS, qSOFA, and SOFA, and a threshold of 5 or more points for NEWS. Glasgow coma scale (GCS) ≤14 was used to define altered mental status [10, 28]. No contribution was made to the total score if an individual component of the score was missing. Patients in whom all components of any score were missing were excluded from analysis.

### Outcomes

The primary outcome was prognostic accuracy of individual scores for hospital mortality. We did not compare the ability of scores to identify patients with sepsis because of the limitations of the retrospective study design and the lack of a gold standard for comparison. Secondary outcomes included hospital mortality stratified by non-ICU and ICU setting at time of inclusion, transfer to the ICU from a non-ICU setting, ICU LOS >3 days, and overall hospital LOS >7 days following study inclusion. ICU transfer was further defined as patient transfer from a non-ICU to ICU setting within the 24 hours preceding and the 24 hours following time of study inclusion with subsequent ICU duration of at least 24 hours or death within 24 hours of transfer. Secondary outcomes were chosen to reflect clinical events significant to both individual patients and more broadly to hospitals and health systems.

### Statistical analysis

All analyses were performed using Stata version 15 (StataCorp, College Station, TX). Patient characteristics are presented as number (%), mean ± standard deviation (SD) for quantification of normally distributed variables, or median and interquartile range (IQR) for non-normally distributed variables. For comparison of continuous variables, Student’s t-test was used. For comparison of dichotomous variables, chi-square test was applied. Comparative prognostic accuracy for the primary and secondary outcomes was assessed using the area under the receiver operating characteristic curve (AUROC) for each score individually (crude analysis) and in conjunction with a baseline risk model (adjusted analysis) to demonstrate the additional prognostic value of sepsis scores beyond potentially confounding demographic factors. Age, sex, and race were used to calculate a baseline level of risk for mortality and other outcomes based on sepsis data from the United States demonstrating disparities according to these factors [29–31]. Adjusted risk ratios for outcomes comparing positive vs. negative scores (e.g., SOFA >2 vs. SOFA <2) were assessed. A 2-sided p-value of <0.01 was used to indicate statistical significance and ensure a robust analysis based on the Bonferroni correction for multiple comparisons [32].

## Results

There were 125,431 patient encounters during the study period, of which 10,981 met study inclusion criteria. Thirty-nine patients had incomplete records precluding the calculation of at least one of the scores and were omitted from analysis; 10,942 patients were included in the study (S1 Fig). Median age was 52 years (IQR, 29–75 years), 70% were male (n = 7,645), and 30% were female (n = 3,297) (Table 1). 61% were Caucasian (n = 6,710), 18% African-American (n = 1,922), and 21% other racial/ethnic groups (n = 2,349). 7,193 patients (66%) were in a non-ICU setting at the time they met inclusion criteria, compared to 3,749 (34%) in the ICU. 774 patients (7.1%) died in the hospital; of these, 116 (15%) deaths occurred in a non-ICU setting and 658 (85%) occurred in the ICU. 313 patients were transferred from a non-ICU setting to the ICU. Of those patients who were admitted to the ICU at any point during their hospitalization, 1,817 patients (48%) had an ICU LOS > 3 days. Hospital LOS >7 days occurred in 3,428 patients (31%).

**Table 1:** Demographics and score distributions among patients hospitalized with suspected infection.

Variable	All	Survivors	Non-survivors
**Patients, N (%)**	10942 (100)	10164 (93)	774 (7.1)
**Age, median (IQR)**	52 (29–75)	52 (30–74)	62 (41–83)
**Male gender, n (%)**	7645 (70)	7099 (70)	546 (70)
**Race, n (%)**			
White	6710 (61)	6250 (62)	460 (59)
Black	1922 (18)	1796 (18)	126 (16)
Other	2310 (21)	2118 (21)	192 (25)
**Location at time of inclusion, n (%)**			
Non-ICU	7193 (66)	7077 (70)	116 (15)
ICU	3749 (34)	3091 (30)	658 (85)
**SIRS ≥2, n (%)**	8534 (78)	7785 (77)	749 (96)
**SIRS, median (IQR)**	3 (2–4)	2 (1–3)	4 (3–4)
**qSOFA ≥2, n (%)**	4864 (45)	4157 (41)	707 (90)
**qSOFA, median (IQR)**	1 (0–2)	1 (0–2)	3 (2–3)
**NEWS ≥5, n (%)**	9746 (89)	8971 (88)	775 (99)
**NEWS, median (IQR)**	9 (2–16)	9 (2–16)	15 (12–18)
**SOFA ≥2, n (%)**	6219 (57)	5473 (55)	746 (96)
**SOFA, median (IQR)**	2 (0–6)	2 (0–6)	10 (2–18)
**Outcomes, n (%)**			
Hospital Mortality	774 (7.1)	-	774 (100)
Non-ICU Mortality^1^	116 (1.6)	-	116 (100)
ICU Mortality^2^	658 (17.6)	-	658 (100)
ICU Transfer^1^	313 (4.4)	257 (3.6)	56 (47)
ICU LOS >3d^2^	1817 (48)	1444 (47)	373 (57)
LOS >7d	3428 (31)	3182 (31)	246 (31)

Abbreviations: ICU, intensive care unit; IQR, interquartile range; LOS, length of stay; NEWS, national early warning score; qSOFA, quick sequential organ failure assessment; SIRS, systemic inflammatory response syndrome; SOFA, sequential organ failure assessment.

^1^ Denominator is N corresponding to non-ICU population for each column.

^2^ Denominator is N corresponding ICU population for each column.

**Fig 1:**
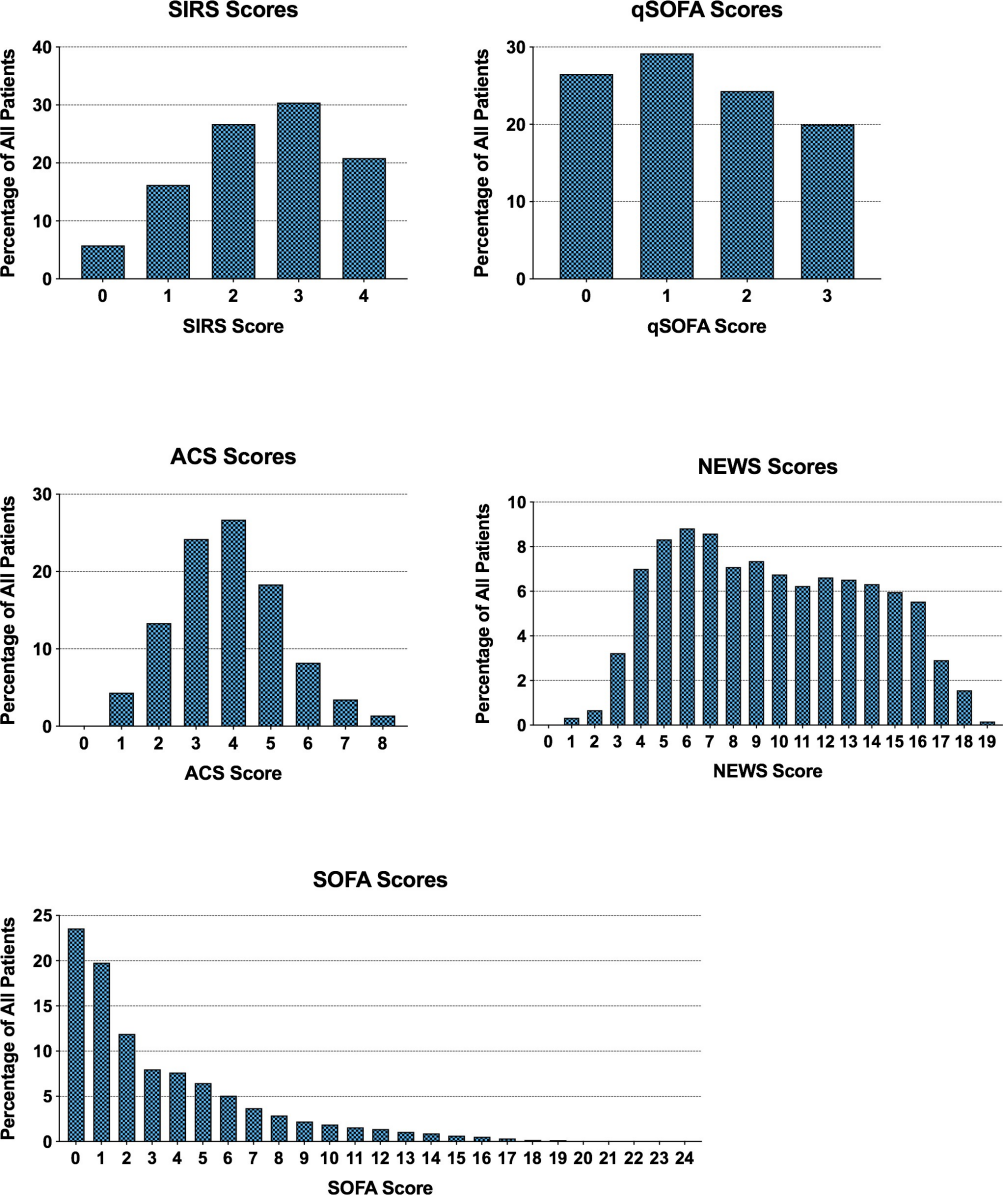
Distribution of patients by scores among adult patients with suspected infection. Abbreviations: ICU, intensive care unit; NEWS, national early warning system score; qSOFA, quick sequential organ function assessment; SIRS, systemic inflammatory response syndrome; SOFA, sequential organ function assessment. Number of patients included in the analysis were 10,942 for all scores. Bolded portion of Y-axis scale indicates the range from 0% to 10%.

**Fig 2:**
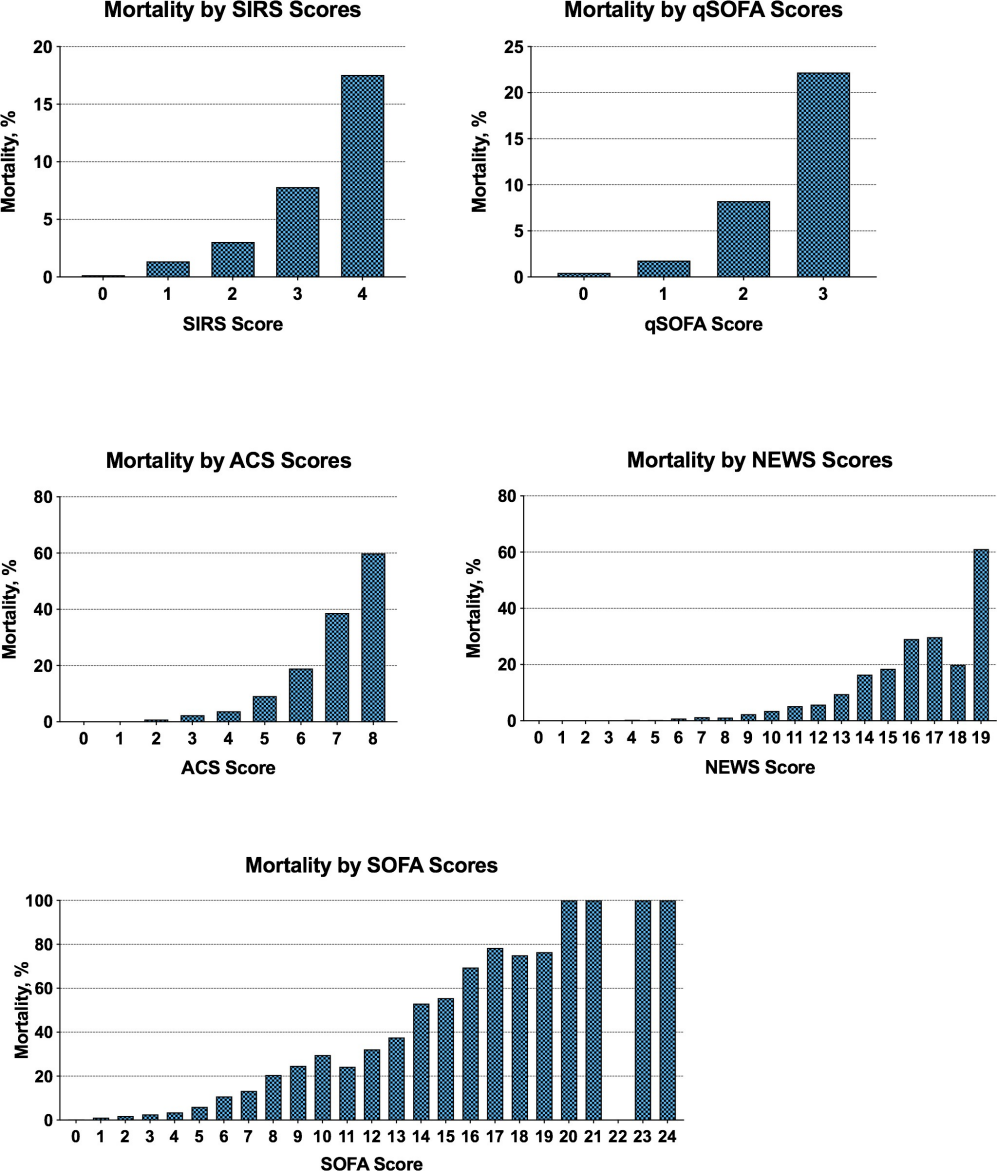
Hospital mortality by score among adult patients with suspected infection. Abbreviations: ICU, intensive care unit; NEWS, national early warning system score; qSOFA, quick sequential organ function assessment; SIRS, systemic inflammatory response syndrome; SOFA, sequential organ function assessment. Number of patients included in the analysis were 10,942 for all scores. Bolded portion of Y-axis scale indicates the range from 0% to 25%.

Within the study cohort, 8,534 (78%) had a SIRS score ≥2; 4,864 (44%) had a qSOFA score ≥2; 9,746 (89%) had a NEWS score ≥5; and 6,219 (57%) had a SOFA score of ≥2. The full distributions of scores and their relationship with hospital mortality are presented in Figs 1 and 2. The incidence of missing score components in the study cohort was low (S1 Table). However, the PaO2/FiO2 ratio and serum bilirubin level were missing for 77% (n = 8,489) and 36% (n = 3,927) of patients, respectively.

After adjusting for baseline risk factors for death, discrimination of hospital mortality was significantly higher for SOFA (AUROC 0.90 [95% CI, 0.89–0.91]) than NEWS (AUROC 0.85 [95% CI, 0.84–0.86], p<0.001), qSOFA (AUROC 0.84 [95% CI, 0.83–0.85], p<0.001), or SIRS (AUROC 0.79 [95% CI, 0.78–0.81], p<0.001) (Table 2, S2 Fig). Similarly, SOFA (AUROC 0.82 [95% CI, 0.80–0.83]) outperformed all other scores in discrimination of hospital mortality in an ICU setting (NEWS AUROC 0.70 [95% CI, 0.68–0.72]; qSOFA AUROC 0.68 [95% CI, 0.66–0.70]; SIRS AUROC 0.68 [95% CI, 0.66–0.70]; all p<0.001) (S3 Fig). For hospital mortality in a non-ICU setting SOFA (AUROC 0.86 [95% CI, 0.83–0.89]) and NEWS (AUROC 0.84 [95% CI, 0.81–0.88]) had similar prognostic accuracy (p = 0.38) and were no different from qSOFA (AUROC 0.84 [95% CI, 0.80–0.87]; qSOFA vs. NEWS p = 0.26; qSOFA vs. SOFA p = 0.129) but were more predictive than SIRS (AUROC 0.81 [95% CI, 0.78–0.84]; SIRS vs. NEWS p = 0.012; SIRS vs. SOFA p = 0.004) (S4 Fig).

**Table 2:** Adjusted AUROCs and comparisons for prediction of hospital mortality outcomes.

Outcome	SIRS	qSOFA	NEWS	SOFA
**Mortality (N = 10942)** AUROC (95% CI)	0.79 (0.78–0.81)	0.84 (0.83–0.85)	0.85 (0.84–0.86)	0.90 (0.89–0.91)
*vs*. *SIRS*		<0.001	<0.001	<0.001
*vs*. *qSOFA*	<0.001		<0.001	<0.001
*vs*. *NEWS*	<0.001	<0.001		<0.001
*vs*. *SOFA*	<0.001	<0.001	<0.001	
**ICU Mortality (n = 3749)** AUROC (95% CI)	0.68 (0.66–0.70)	0.68 (0.66–0.70)	0.70 (0.68–0.72)	0.82 (0.80–0.83)
*vs*. *SIRS*		0.84	0.03	<0.001
*vs*. *qSOFA*	0.84		0.003	<0.001
*vs*. *NEWS*	0.03	0.003		<0.001
*vs*. *SOFA*	<0.001	<0.001	<0.001	
**Non-ICU Mortality (n = 7193)** AUROC (95% CI)	0.81 (0.78–0.84)	0.84 (0.80–0.87)	0.84 (0.81–0.88)	0.86 (0.83–0.89)
*vs*. *SIRS*		0.08	0.01	0.004
*vs*. *qSOFA*	0.08		0.26	0.13
*vs*. *NEWS*	0.01	0.26		0.38
*vs*. *SOFA*	0.004	0.13	0.38	

Abbreviations: AUROC, area under the receiver operator curve; CI, confidence interval; ICU, intensive care unit; NEWS, national early warning score; qSOFA, quick sequential organ failure assessment; SIRS, systemic inflammatory response syndrome; SOFA, sequential organ failure assessment. N values correspond to the number of patients included in the analysis who were eligible to experience the outcome.

NEWS (AUROC 0.94 [95% CI, 0.93–0.95]) outperformed qSOFA (AUROC 0.89 [95% CI, 0.87–0.91], p<0.001), SOFA (AUROC 0.84, [95% CI, 0.82–0.87], p<0.001), and SIRS (AUROC 0.81 [95% CI, 0.79–0.83], p<0.001) in predicting transfer from a non-ICU to an ICU setting (Table 3; S5 Fig). Similarly, NEWS (AUROC 0.86 [95% CI, 0.85–0.86]) was superior to SOFA (AUROC 0.84 [95% CI, 0.83–0.85], p = 0.002), qSOFA (AUROC 0.83 [95% CI, 0.83–0.84], p<0.001), and SIRS (AUROC 0.75 [95% CI, 0.74–0.76], p<0.001) in predicting ICU LOS >3 days (S6 Fig). For hospital LOS >7 days qSOFA (AUROC 0.64 [95% CI, 0.63–0.65]), NEWS (AUROC 0.65 [95% CI, 0.64–0.66]), and SOFA (AUROC 0.64 [95% CI, 0.63–0.65]) were all more predictive than SIRS (SIRS vs. qSOFA p = 0.002; SIRS vs. NEWS p<0.001; SIRS vs. SOFA p = 0.01) but were not statistically different from each other (S7 Fig).

**Table 3:** Adjusted AUROCs and comparisons for prediction of ICU transfer and length of stay outcomes.

Outcome	SIRS	qSOFA	NEWS	SOFA
**ICU Transfer (n = 7287)** AUROC (95% CI)	0.81 (0.79–0.83)	0.89 (0.87–0.91)	0.94 (0.93–0.95)	0.84 (0.82–0.87)
*vs*. *SIRS*		<0.001	<0.001	0.03
*vs*. *qSOFA*	<0.001		<0.001	<0.001
*vs*. *NEWS*	<0.001	<0.001		<0.001
*vs*. *SOFA*	0.03	<0.001	<0.001	
**ICU LOS >3 days (N = 10942)** AUROC (95% CI)	0.75 (0.74–0.76)	0.83 (0.83–0.84)	0.86 (0.85–0.86)	0.84 (0.83–0.85)
*vs*. *SIRS*		<0.001	<0.001	<0.001
*vs*. *qSOFA*	<0.001		<0.001	0.11
*vs*. *NEWS*	<0.001	<0.001		0.002
*vs*. *SOFA*	<0.001	0.11	0.002	
**LOS >7 days (N = 10942)** AUROC (95% CI)	0.63 (0.61–0.64)	0.64 (0.63–0.65)	0.65 (0.64–0.66)	0.64 (0.63–0.65)
*vs*. *SIRS*		0.002	<0.001	0.01
*vs*. *qSOFA*	0.002		0.03	0.52
*vs*. *NEWS*	<0.001	0.03		0.08
*vs*. *SOFA*	0.01	0.52	0.08	

Abbreviations: AUROC, area under the receiver operator curve; CI, confidence interval; ICU, intensive care unit; NEWS, national early warning score; quick sequential organ failure assessment; SIRS, systemic inflammatory response syndrome; SOFA, sequential organ failure assessment. N values correspond to the number of patients included in the analysis who were eligible to experience the outcome. For ICU transfer, the reported n of 7,287 indicates the 7193 non-ICU patients and an additional 94 patients who were in the ICU at time of inclusion but had been transferred to the ICU within the preceding 24 hours and met the definition for ICU transfer.

Analyses using crude data without adjustment for baseline risk of mortality are reported in the supplement and resulted in similar estimates of AUROC for outcomes by each scoring system (S2 Table, S3 Table, S8–S13 Figs).

Assessment of adjusted risk ratios for outcomes comparing positive vs. negative scores in a binary fashion (e.g., qSOFA ≥2 vs. qSOFA <2) are also reported in the supplement (S14 Fig). Relative risk of hospital mortality (RR 47.0 [95% CI, 11.7–188.8]), non-ICU mortality (RR 10.0 [95% CI, 2.5–40.6]), ICU transfer (RR 51.0 [95% CI, 31.1–83.5]), and ICU LOS >3 days (RR 67.4 [95% CI, 25.2–180.1]) were highest with NEWS ≥5. SOFA ≥2 (RR 10.9 [95% CI, 5.4–22.2]) had the highest relative risk of ICU mortality. There was no appreciable difference in the relative risk of hospital LOS >7 days between scores.

## Discussion

In this study comparing the prognostic accuracy of SOFA, qSOFA, SIRS, and NEWS scores for clinically relevant outcomes in a large population of adult patients with suspected infection at an academic public hospital, more detailed multivariate prediction scores outperformed simpler scores. SOFA, a complex score combining physiologic and laboratory data, demonstrated superior prognostic accuracy for overall hospital mortality and ICU mortality compared to all other scores. NEWS, which utilizes physiologic data only, outperformed all other scores in predicting transfer to the ICU and for ICU LOS >3 days. There was no significant difference between qSOFA and SIRS in predicting the primary outcome of hospital mortality. However, qSOFA was superior to SIRS in predicting transfer to the ICU, ICU LOS >3 days, and hospital LOS >7 days in the study cohort.

This study’s finding that SIRS is a poor predictor of mortality is consistent with prior studies [33, 34]. In the sepsis consensus definition paper, the ability of SOFA, qSOFA, and SIRS to predict hospital mortality was determined in a mixed cohort of ICU and non-ICU encounters [10]. Seymour and colleagues found that 1) the predictive validity of qSOFA for hospital mortality was statistically greater than SOFA or SIRS for non-ICU encounters, and 2) the predictive validity of SOFA for hospital mortality was superior to qSOFA or SIRS for ICU encounters [10]. Freund et al. conducted an international prospective cohort study that showed the superiority of qSOFA in prognosticating hospital mortality as compared to SIRS in patients presenting to the emergency department with suspected infection [13]. Raith et al. performed a retrospective cohort analysis on a large cohort of patients in Australian and New Zealand ICUs and found that SOFA had greater prognostic accuracy for hospital mortality compared to SIRS or qSOFA [14]. Moreover, qSOFA was found to have superior discrimination of mortality as compared to SIRS in adult patients with suspected infection hospitalized in low- and middle-income countries [15]. A recent meta-analysis of 38 studies comparing the prognostic accuracy of qSOFA and SIRS for hospital mortality among patients with suspected infection reported that qSOFA was more predictive of mortality but SIRS was superior for sepsis diagnosis [22]. However, these findings are inconclusive given that the included studies varied significantly in the studied patient population (e.g., ED vs. acute care vs. ICU), outcome measures (hospital mortality vs. 28-day mortality), and on the definition of “suspected infection,” with only 10 of the 38 studies using a standardized approach incorporating antibiotic treatment or initiation of body fluid cultures.

This study found that SOFA outperformed qSOFA, SIRS, and NEWS in predicting overall hospital mortality and ICU mortality. Nonetheless, the observed event rate of non-ICU fatalities was low in this cohort. Given that the majority of deaths occurred in the ICU, we found few significant differences between scores in prognosticating non-ICU mortality. This study further confirms the superiority of SOFA to discriminate ICU mortality, as previously reported by others [14, 16]. In this cohort qSOFA was superior to SIRS in predicting overall hospital mortality, transfer to the ICU, ICU LOS >3 days, and hospital LOS >7 days. This finding is consistent with the meta-analysis by Fernando et al. in which qSOFA was found to be superior to SIRS in predicting hospital mortality [22]. However, we found that NEWS had better discriminative value than qSOFA for hospital mortality and was superior to all other scores in predicting ICU transfer and ICU LOS >3 days. This finding strongly supports mounting evidence that qSOFA should not replace general early-warning scores in risk-stratifying patients with suspected infection in high-resource hospitalized settings [20, 23, 24, 26]. However, qSOFA may have utility in environments where calculation of complex scores is a challenge, such as outpatient clinics, emergency departments, or low-resource settings.

This study has several strengths. The analysis is based on a large dataset encompassing patients in non-ICU and ICU settings and includes both medical and surgical patient cohorts. The dataset was designed to address the study question and was further strengthened by the low incidence of missing data elements. The data have excellent external validity as evidenced by a hospital mortality rate of 1.7% in the non-ICU cohort and 16.4% in the ICU cohort that is highly consistent with other published reports [10, 14]. This work benefitted from its use of a reproducible identification schema for suspected infection and use of similar methodology to the consensus paper and other major studies [7, 10, 14, 15, 23]. Hospital mortality was the primary outcome, but the study also measured the ability of scores to prognosticate other clinically relevant outcomes pertinent to individual patients, hospitals, and healthcare systems that have not been previously reported in other large studies of this kind.

Data were retrospectively collected for this analysis and were limited to a single academic public hospital in the United States. The majority of patients in this cohort were male and/or Caucasian. While this is consistent with the hospital’s overall patient demographics and previously published studies from this center, the generalizability of the results to other hospitals and healthcare settings with different patient demographics is unclear [35]. Calculation of scores was based upon the most deranged physiologic and biochemical score components within the 24 hours preceding and 24 hours following inclusion in the study, consistent with other studies in the field [7, 10, 14, 15, 23]. Thus, these data may bias towards higher scores. Additionally, missing data regarding the respiratory and hepatic components of the SOFA score may have limited its prognostic accuracy for mortality outcomes in this analysis. While adjusted analyses were performed to demonstrate the additive power of sepsis scores to predict outcomes beyond baseline risk, the variables used to generate this model were limited in scope due to lack of administrative data pertaining to relevant comorbid conditions on admission. This study reports on the performance of scores in ICU and non-ICU settings but does not compare score performance across the ED, acute care service, and ICU due to its retrospective nature and limitations of the available administrative data. Finally, the majority of deaths in this study cohort occurred in the ICU and thus the present work may be underpowered to determine which score performs best in non-ICU environments.

## Conclusions

In this large single-center retrospective cohort study of adult medical and surgical inpatients with suspected infection, multivariate prediction scores such as SOFA and NEWS demonstrated superior prognostic accuracy for hospital mortality, ICU transfer, and ICU LOS as compared to qSOFA and SIRS. These findings suggest that complex scores may such as SOFA and NEWS may offer enhanced prognostic performance over simple sepsis scores such as qSOFA and SIRS in inpatient hospital settings where more complex scores can be readily calculated.

## Supporting information

S1 Table Missing score components in the final study cohort. Abbreviations: FiO2, fraction of inspired oxygen; GCS, Glasgow coma scale; ICU, intensive care unit; MAP, mean arterial pressure; NEWS, national early warning score; O2, oxygen; PaO2, partial pressure of arterial oxygen; qSOFA, quick sequential organ failure assessment; SBP, systolic blood pressure; SIRS, systemic inflammatory response syndrome; SOFA, sequential organ failure assessment; WBC, white blood cell count.
(DOCX)

S2 Table Crude AUROCs and comparisons for prediction of hospital mortality outcomes. Abbreviations: AUROC, area under the operator receiver curve; CI, confidence interval; ICU, intensive care unit; NEWS, national early warning score; qSOFA, quick sequential organ failure assessment; SIRS, systemic inflammatory response syndrome; SOFA, sequential organ failure assessment. N values correspond to the number of patients included in the analysis who were eligible to experience the outcome.
(DOCX)

S3 Table Crude AUROCs and comparisons for prediction of ICU transfer and length of stay outcomes. Abbreviations: AUROC, area under the operator receiver curve; CI, confidence interval; ICU, intensive care unit; NEWS, national early warning score; qSOFA, quick sequential organ failure assessment; SIRS, systemic inflammatory response syndrome; SOFA, sequential organ failure assessment. N values correspond to the number of patients included in the analysis who were eligible to experience the outcome. For ICU transfer, the reported n of 7,287 indicates the 7193 non-ICU patients and an additional 94 patients who were in the ICU at time of inclusion but had been transferred to the ICU within the preceding 24 hours and met the definition for ICU transfer.
(DOCX)

S1 Fig Flow diagram of eligible patient population and exclusion criteria. Abbreviations: ICU, intensive care unit. 125,431 patient encounters were screened for eligibility. Following exclusion of patients <18 years of age, patients who were directly admitted to the hospital or were transferred from outside institutions, were admitted to inpatient psychiatric or rehabilitation services, were evaluated in the ED and discharged, or encounters did not meet criteria for suspected infection, 10,981 patients remained. A further 39 patients (4 ICU and 35 non-ICU) were omitted from the final study cohort because all components of one of the sepsis scores were missing. The final study cohort comprised 10,942 patient encounters.
(TIFF)

S2 Fig Adjusted area under the receiver operating characteristic curves (AUROCs) of prognostic accuracy for in-hospital mortality for SIRS, qSOFA, NEWS, and SOFA scores. Abbreviations: NEWS, national early warning score; qSOFA, quick sequential organ function assessment; SIRS, systemic inflammatory response syndrome; SOFA, sequential organ function assessment. Adjusted AUROCs: SOFA, 0.90 (95% CI, 0.89–0.91); NEWS, 0.85 (95% CI, 0.84–0.86); qSOFA, 0.84 (95% CI, 0.83–0.85); SIRS, 0.79 (95% CI, 0.78–0.81); model of baseline risk, 0.67 (95% CI, 0.65–0.69).
(TIF)

S3 Fig Adjusted area under the receiver operating characteristic curves (AUROCs) of prognostic accuracy for ICU mortality for SIRS, qSOFA, NEWS, and SOFA scores. Abbreviations: NEWS, national early warning score; qSOFA, quick sequential organ function assessment; SIRS, systemic inflammatory response syndrome; SOFA, sequential organ function assessment. Adjusted AUROCs: SOFA, 0.82 (95% CI, 0.80–0.83); NEWS, 0.70 (95% CI, 0.68–0.72); qSOFA, 0.68 (95% CI, 0.66–0.70); SIRS, 0.68 (95% CI, 0.66–0.70); model of baseline risk, 0.62 (95% CI, 0.59–0.64).
(TIF)

S4 Fig Adjusted area under the receiver operating characteristic curves (AUROCs) of prognostic accuracy for non-ICU mortality for SIRS, qSOFA, NEWS, and SOFA scores. Abbreviations: NEWS, national early warning score; qSOFA, quick sequential organ function assessment; SIRS, systemic inflammatory response syndrome; SOFA, sequential organ function assessment. Adjusted AUROCs: SOFA, 0.86 (95% CI, 0.83–0.89); NEWS, 0.84 (95% CI, 0.81–0.88); qSOFA, 0.84 (95% CI, 0.80–0.87); SIRS, 0.81 (95% CI, 0.78–0.84); model of baseline risk, 0.77 (95% CI, 0.74–0.81).
(TIF)

S5 Fig Adjusted area under the receiver operating characteristic curves (AUROCs) of prognostic accuracy for ICU transfer for SIRS, qSOFA, NEWS, and SOFA scores. Abbreviations: NEWS, national early warning score; qSOFA, quick sequential organ function assessment; SIRS, systemic inflammatory response syndrome; SOFA, sequential organ function assessment. Adjusted AUROCs: NEWS, 0.94 (95% CI, 0.93–0.95); qSOFA, 0.89 (95% CI, 0.87–0.91); SOFA, 0.84 (95% CI, 0.82–0.87); SIRS, 0.81 (95% CI, 0.79–0.83); model of baseline risk, 0.64 (95% CI, 0.61–0.67).
(TIF)

S6 Fig Adjusted area under the receiver operating characteristic curves (AUROCs) of prognostic accuracy for ICU length of stay (LOS) >3 days for SIRS, qSOFA, NEWS, and SOFA scores. Abbreviations: NEWS, national early warning score; qSOFA, quick sequential organ function assessment; SIRS, systemic inflammatory response syndrome; SOFA, sequential organ function assessment. Adjusted AUROCs: NEWS, 0.86 (95% CI, 0.85–0.86); SOFA, 0.84 (95% CI, 0.83–0.85); qSOFA, 0.83 (95% CI, 0.83–0.84); SIRS, 0.75 (95% CI, 0.74–0.76); model of baseline risk, 0.58 (95% CI, 0.57–0.60).
(TIF)

S7 Fig Adjusted area under the receiver operating characteristic curves (AUROCs) of prognostic accuracy for hospital length of stay (LOS) >7 days for SIRS, qSOFA, NEWS, and SOFA scores. Abbreviations: NEWS, national early warning score; qSOFA, quick sequential organ function assessment; SIRS, systemic inflammatory response syndrome; SOFA, sequential organ function assessment. Adjusted AUROCs: qSOFA, 0.64 (95% CI, 0.63–0.65); NEWS, 0.65 (95% CI, 0.64–0.66); SOFA, 0.64 (95% CI, 0.63–0.65); SIRS, 0.63 (95% CI, 0.61–0.64); model of baseline risk, 0.56 (95% CI, 0.55–0.57).
(TIF)

S8 Fig Crude area under the receiver operating characteristic curves (AUROCs) of prognostic accuracy for hospital mortality for SIRS, qSOFA, NEWS, and SOFA scores. Abbreviations: NEWS, national early warning score; qSOFA, quick sequential organ function assessment; SIRS, systemic inflammatory response syndrome; SOFA, sequential organ function assessment. Crude AUROCs: SOFA 0.88 (95% CI, 0.87–0.90); NEWS, 0.84 (95% CI, 0.83–0.85); qSOFA, 0.81 (95% CI, 0.80–0.82); SIRS, 0.74 (95% CI, 0.72–0.76).
(TIF)

S9 Fig Crude area under the receiver operating characteristic curves (AUROCs) of prognostic accuracy for ICU mortality for SIRS, qSOFA, NEWS, and SOFA scores. Abbreviations: NEWS, national early warning score; qSOFA, quick sequential organ function assessment; SIRS, systemic inflammatory response syndrome; SOFA, sequential organ function assessment. Crude AUROCs: SOFA, 0.80 (95% CI, 0.78–0.82); NEWS, 0.67 (95% CI, 0.65–0.70); qSOFA, 0.63 (95% CI, 0.61–0.65); SIRS, 0.62 (95% CI, 0.60–0.64).
(TIF)

S10 Fig Crude area under the receiver operating characteristic curves (AUROCs) of prognostic accuracy for non-ICU mortality for SIRS, qSOFA, NEWS, and SOFA scores. Abbreviations: NEWS, national early warning score; qSOFA, quick sequential organ function assessment; SIRS, systemic inflammatory response syndrome; SOFA, sequential organ function assessment. Crude AUROCs: SOFA, 0.80 (95% CI, 0.75–0.84); NEWS, 0.77 (95% CI, 0.73–0.81); qSOFA, 0.75 (95% CI, 0.71–0.79); SIRS, 0.67 (95% CI, 0.62–0.72).
(TIF)

S11 Fig Crude area under the receiver operating characteristic curves (AUROCs) of prognostic accuracy for ICU transfer for SIRS, qSOFA, NEWS, and SOFA scores. Abbreviations: NEWS, national early warning score; qSOFA, quick sequential organ function assessment; SIRS, systemic inflammatory response syndrome; SOFA, sequential organ function assessment. Crude AUROCs: NEWS, 0.94 (95% CI, 0.93–0.95); qSOFA, 0.89 (95% CI, 0.87–0.90); SOFA, 0.84 (95% CI, 0.81–0.86); SIRS, 0.77 (95% CI, 0.75–0.79).
(TIF)

S12 Fig Crude area under the receiver operating characteristic curves (AUROCs) of prognostic accuracy for ICU length of stay (LOS) >3 days for SIRS, qSOFA, NEWS, and SOFA scores. Abbreviations: NEWS, national early warning score; qSOFA, quick sequential organ function assessment; SIRS, systemic inflammatory response syndrome; SOFA, sequential organ function assessment. Crude AUROCs: NEWS, 0.85 (95% CI, 0.85–0.86); SOFA, 0.84 (95% CI, 0.83–0.85); qSOFA, 0.83 (95% CI, 0.82–0.84); SIRS, 0.73 (95% CI, 0.72–0.74).
(TIF)

S13 Fig Crude area under the receiver operating characteristic curves (AUROCs) of prognostic accuracy for hospital length of stay (LOS) >7 days for SIRS, qSOFA, NEWS, and SOFA scores. Abbreviations: NEWS, national early warning score; qSOFA, quick sequential organ function assessment; SIRS, systemic inflammatory response syndrome; SOFA, sequential organ function assessment. Crude AUROCs: qSOFA, 0.63 (95% CI, 0.62–0.64); NEWS, 0.64 (95% CI, 0.63–0.65); SOFA, 0.63 (95% CI, 0.62–0.64); SIRS, 0.61 (95% CI, 0.60–0.62).
(TIF)

S14 Fig Adjusted risk ratios for outcomes comparing positive vs. negative SIRS, qSOFA, NEWS, and SOFA scores. Abbreviations: ICU, intenstive care unity; LOS, length of stay; NEWS, national early warning score; qSOFA, quick sequential organ function assessment; RR, risk ratio; SIRS, systemic inflammatory response syndrome; SOFA, sequential organ function assessment. Criteria for score positivity: SIRS ≥2, qSOFA ≥2, NEWS ≥5, SOFA ≥2. A) Adjusted risk ratios for hospital mortality: NEWS, 47.0 (95% CI, 11.7–188.8), SOFA, 19.2 (95% CI, 13.1–28.1); qSOFA, 13.3 (95% CI, 10.3–17.2); SIRS, 9.0 (95% CI, 6.0–13.5). B) Adjusted risk ratios for ICU mortality: SOFA, 10.9 (95% CI, 5.4–22.2); qSOFA, 5.5 (95% CI, 3.5–8.1); SIRS, 3.9 (95% CI, 2.0–6.6). No patients with NEWS <5 died in the ICU and thus risk ratio of NEWS for ICU mortality could not be calculated; therefore NEWS is omitted from panel B. C) Adjusted risk ratios for non-ICU mortality: NEWS, 10.0 (95% CI, 2.5–40.6); SOFA, 5.0 (95% CI, 3.0–8.2); qSOFA, 3.8 (95% CI, 2.6–5.6); SIRS, 3.1 (95% CI, 1.8–5.4). D) Adjusted risk ratios for ICU transfer: NEWS, 51.0 (95% CI, 31.1–83.5); qSOFA, 20.5 (95% CI, 14.9–28.3); SIRS, 14.3 (95% CI, 7.6–27.0); SOFA, 9.1 (95% CI, 6.5–12.7). E) Adjusted risk ratios for ICU LOS >3 days: NEWS, 67.4 (95% CI, 25.2–180.1); qSOFA, 16.6 (95% CI, 14.1–19.5); SOFA, 12.4 (95% CI, 10.3–14.9); SIRS, 9.4 (95% CI, 7.3–12.1). F) Adjusted risk ratios for hospital LOS >7 days: qSOFA, 2.4 (95% CI, 2.2–2.6); SOFA, 2.1 (95% CI, 2.0–2.3); SIRS, 2.1 (95% CI, 1.9–2.4); NEWS, 2.1 (95% CI, 1.8–2.4). Error bars represent 95% confidence interval.
(TIFF)

## References

[1] Paoli CJ, Reynolds MA, Sinha M, Gitlin M, Crouser E. Epidemiology and Costs of Sepsis in the United States-An Analysis Based on Timing of Diagnosis and Severity Level. Crit Care Med. 2018;46(12):1889–97. 10.1097/CCM.0000000000003342 30048332 PMC6250243

[2] Liu V, Escobar GJ, Greene JD, Soule J, Whippy A, Angus DC, et al Hospital deaths in patients with sepsis from 2 independent cohorts. Jama. 2014;312(1):90–2. 10.1001/jama.2014.5804 24838355

[3] Martin GS, Mannino DM, Eaton S, Moss M. The epidemiology of sepsis in the United States from 1979 through 2000. N Engl J Med. 2003;348(16):1546–54. 10.1056/NEJMoa022139 12700374

[4] Rudd KE, Delaney A, Finfer S. Counting Sepsis, an Imprecise but Improving Science. Jama. 2017;318(13):1228–9. 10.1001/jama.2017.13697 28903164

[5] Fleischmann C, Scherag A, Adhikari NK, Hartog CS, Tsaganos T, Schlattmann P, et al Assessment of Global Incidence and Mortality of Hospital-treated Sepsis. Current Estimates and Limitations. Am J Respir Crit Care Med. 2016;193(3):259–72. 10.1164/rccm.201504-0781OC 26414292

[6] Hatfield KM, Dantes RB, Baggs J, Sapiano MRP, Fiore AE, Jernigan JA, et al Assessing Variability in Hospital-Level Mortality Among U.S. Medicare Beneficiaries With Hospitalizations for Severe Sepsis and Septic Shock. Crit Care Med. 2018;46(11):1753–60. 10.1097/CCM.0000000000003324 30024430 PMC6774245

[7] Rhee C, Dantes R, Epstein L, Murphy DJ, Seymour CW, Iwashyna TJ, et al Incidence and Trends of Sepsis in US Hospitals Using Clinical vs Claims Data, 2009–2014. Jama. 2017;318(13):1241–9. 10.1001/jama.2017.13836 28903154 PMC5710396

[8] Yealy DM, Huang DT, Delaney A, Knight M, Randolph AG, Daniels R, et al Recognizing and managing sepsis: what needs to be done? BMC Med. 2015;13(98):015–0335. 10.1186/s12916-015-0335-2 PMC4410741 25927426

[9] Seymour CW, Gesten F, Prescott HC, Friedrich ME, Iwashyna TJ, Phillips GS, et al Time to Treatment and Mortality during Mandated Emergency Care for Sepsis. N Engl J Med. 2017;376(23):2235–44. 10.1056/NEJMoa1703058 28528569 PMC5538258

[10] Seymour CW, Liu VX, Iwashyna TJ, Brunkhorst FM, Rea TD, Scherag A, et al Assessment of Clinical Criteria for Sepsis: For the Third International Consensus Definitions for Sepsis and Septic Shock (Sepsis-3). Jama. 2016;315(8):762–74. 10.1001/jama.2016.0288 26903335 PMC5433435

[11] Vincent JL, de Mendonca A, Cantraine F, Moreno R, Takala J, Suter PM, et al Use of the SOFA score to assess the incidence of organ dysfunction/failure in intensive care units: results of a multicenter, prospective study. Working group on "sepsis-related problems" of the European Society of Intensive Care Medicine. Crit Care Med. 1998;26(11):1793–800. 10.1097/00003246-199811000-00016 9824069

[12] Bone RC, Balk RA, Cerra FB, Dellinger RP, Fein AM, Knaus WA, et al Definitions for sepsis and organ failure and guidelines for the use of innovative therapies in sepsis. The ACCP/SCCM Consensus Conference Committee. American College of Chest Physicians/Society of Critical Care Medicine. Chest. 1992;101(6):1644–55. 10.1378/chest.101.6.1644 1303622

[13] Freund Y, Lemachatti N, Krastinova E, Van Laer M, Claessens YE, Avondo A, et al Prognostic Accuracy of Sepsis-3 Criteria for In-Hospital Mortality Among Patients With Suspected Infection Presenting to the Emergency Department. Jama. 2017;317(3):301–8. 10.1001/jama.2016.20329 28114554

[14] Raith EP, Udy AA, Bailey M, McGloughlin S, MacIsaac C, Bellomo R, et al Prognostic Accuracy of the SOFA Score, SIRS Criteria, and qSOFA Score for In-Hospital Mortality Among Adults With Suspected Infection Admitted to the Intensive Care Unit. Jama. 2017;317(3):290–300. 10.1001/jama.2016.20328 28114553

[15] Rudd KE, Seymour CW, Aluisio AR, Augustin ME, Bagenda DS, Beane A, et al Association of the Quick Sequential (Sepsis-Related) Organ Failure Assessment (qSOFA) Score With Excess Hospital Mortality in Adults With Suspected Infection in Low- and Middle-Income Countries. Jama. 2018;319(21):2202–11. 10.1001/jama.2018.6229 29800114 PMC6134436

[16] Minne L, Abu-Hanna A, de Jonge E. Evaluation of SOFA-based models for predicting mortality in the ICU: A systematic review. Crit Care. 2008;12(6):17. 10.1186/cc7160 PMC2646326 19091120

[17] Keegan MT, Gajic O, Afessa B. Severity of illness scoring systems in the intensive care unit. Crit Care Med. 2011;39(1):163–9. 10.1097/CCM.0b013e3181f96f81 20838329

[18] Kollef MH, Schuster DP. Predicting intensive care unit outcome with scoring systems. Underlying concepts and principles. Crit Care Clin. 1994;10(1):1–18. 8118722

[19] Sbiti-Rohr D, Kutz A, Christ-Crain M, Thomann R, Zimmerli W, Hoess C, et al The National Early Warning Score (NEWS) for outcome prediction in emergency department patients with community-acquired pneumonia: results from a 6-year prospective cohort study. BMJ Open. 2016;6(9):2015–011021. 10.1136/bmjopen-2015-011021 PMC5051330 27683509

[20] Goulden R, Hoyle MC, Monis J, Railton D, Riley V, Martin P, et al qSOFA, SIRS and NEWS for predicting inhospital mortality and ICU admission in emergency admissions treated as sepsis. Emerg Med J. 2018;35(6):345–9. 10.1136/emermed-2017-207120 29467173

[21] Churpek MM, Snyder A, Sokol S, Pettit NN, Edelson DP. Investigating the Impact of Different Suspicion of Infection Criteria on the Accuracy of Quick Sepsis-Related Organ Failure Assessment, Systemic Inflammatory Response Syndrome, and Early Warning Scores. Crit Care Med. 2017;45(11):1805–12. 10.1097/CCM.0000000000002648 28737573 PMC5640476

[22] Fernando SM, Tran A, Taljaard M, Cheng W, Rochwerg B, Seely AJE, et al Prognostic Accuracy of the Quick Sequential Organ Failure Assessment for Mortality in Patients With Suspected Infection: A Systematic Review and Meta-analysis. Ann Intern Med. 2018;168(4):266–75. 10.7326/M17-2820 29404582

[23] Churpek MM, Snyder A, Han X, Sokol S, Pettit N, Howell MD, et al Quick Sepsis-related Organ Failure Assessment, Systemic Inflammatory Response Syndrome, and Early Warning Scores for Detecting Clinical Deterioration in Infected Patients outside the Intensive Care Unit. Am J Respir Crit Care Med. 2017;195(7):906–11. 10.1164/rccm.201604-0854OC 27649072 PMC5387705

[24] Szakmany T, Pugh R, Kopczynska M, Lundin RM, Sharif B, Morgan P, et al Defining sepsis on the wards: results of a multi-centre point-prevalence study comparing two sepsis definitions. Anaesthesia. 2018;73(2):195–204. 10.1111/anae.14062 29150856

[25] Prytherch DR, Smith GB, Schmidt PE, Featherstone PI. ViEWS—Towards a national early warning score for detecting adult inpatient deterioration. Resuscitation. 2010;81(8):932–7. 10.1016/j.resuscitation.2010.04.014 20637974

[26] Redfern OC, Smith GB, Prytherch DR, Meredith P, Inada-Kim M, Schmidt PE. A Comparison of the Quick Sequential (Sepsis-Related) Organ Failure Assessment Score and the National Early Warning Score in Non-ICU Patients With/Without Infection. Crit Care Med. 2018;46(12):1923–33. 10.1097/CCM.0000000000003359 30130262

[27] Singer M, Deutschman CS, Seymour CW, Shankar-Hari M, Annane D, Bauer M, et al The Third International Consensus Definitions for Sepsis and Septic Shock (Sepsis-3). Jama. 2016;315(8):801–10. 10.1001/jama.2016.0287 26903338 PMC4968574

[28] Teasdale G, Jennett B. Assessment of coma and impaired consciousness. A practical scale. Lancet. 1974;2(7872):81–4. 10.1016/s0140-6736(74)91639-0 4136544

[29] Nasa P, Juneja D, Singh O, Dang R, Arora V. Severe sepsis and its impact on outcome in elderly and very elderly patients admitted in intensive care unit. J Intensive Care Med. 2012;27(3):179–83. 10.1177/0885066610397116 21436163

[30] Pietropaoli AP, Glance LG, Oakes D, Fisher SG. Gender differences in mortality in patients with severe sepsis or septic shock. Gend Med. 2010;7(5):422–37. 10.1016/j.genm.2010.09.005 21056869 PMC3322379

[31] Chaudhary NS, Donnelly JP, Wang HE. Racial Differences in Sepsis Mortality at U.S. Academic Medical Center-Affiliated Hospitals. Crit Care Med. 2018;46(6):878–83. 10.1097/CCM.0000000000003020 29438109 PMC5953774

[32] Bland JM, Altman DG. Multiple significance tests: the Bonferroni method. Bmj. 1995;310(6973):170 10.1136/bmj.310.6973.170 7833759 PMC2548561

[33] Kaukonen KM, Bailey M, Pilcher D, Cooper DJ, Bellomo R. Systemic inflammatory response syndrome criteria in defining severe sepsis. N Engl J Med. 2015;372(17):1629–38. 10.1056/NEJMoa1415236 25776936

[34] Liao MM, Lezotte D, Lowenstein SR, Howard K, Finley Z, Feng Z, et al Sensitivity of systemic inflammatory response syndrome for critical illness among ED patients. Am J Emerg Med. 2014;32(11):1319–25. 10.1016/j.ajem.2014.07.035 25205616 PMC4254326

[35] Rubenfeld GD, Caldwell E, Peabody E, Weaver J, Martin DP, Neff M, et al Incidence and outcomes of acute lung injury. N Engl J Med. 2005;353(16):1685–93. 10.1056/NEJMoa050333 16236739

