# Correction: Comparative prognostic accuracy of sepsis scores for hospital mortality in adults with suspected infection in non-ICU and ICU at an academic public hospital

**DOI:** 10.1371/journal.pone.0224780

**Published:** 2019-10-30

**Authors:** Christopher P. Kovach, Grant S. Fletcher, Kristina E. Rudd, Rosemary M. Grant, David J. Carlbom

The word "populations" is missing from the article title. The correct title is: Comparative prognostic accuracy of sepsis scores for hospital mortality in adults with suspected infection in non-ICU and ICU populations at an academic public hospital. The correct citation is: Kovach CP, Fletcher GS, Rudd KE, Grant RM, Carlbom DJ (2019) Comparative prognostic accuracy of sepsis scores for hospital mortality in adults with suspected infection in non-ICU and ICU populations at an academic public hospital. PLoS ONE 14(9): e0222563. https://doi.org/10.1371/journal.pone.0222563.
