# Supplementary material for: Comparative prognostic accuracy of sepsis scores for hospital mortality in adults with suspected infection in non-ICU and ICU at an academic public hospital
Source: PLoS One. 2019 Sep 16;14(9):e0222563. doi: 10.1371/journal.pone.0222563 (PMC6746500; doi:10.1371/journal.pone.0222563)
Supplement: S1 Table — Abbreviations: FiO2, fraction of inspired oxygen; GCS, Glasgow coma scale; ICU, intensive care unit; MAP, mean arterial pressure; NEWS, national early warning score; O2, oxygen; PaO2, partial pressure of arterial oxygen; qSOFA, quick sequential organ failure assessment; SBP, systolic blood pressure; SIRS, systemic inflammatory response syndrome; SOFA, sequential organ failure assessment; WBC, white blood cell count. (DOCX) [file pone.0222563.s001.docx]

S1 Table. Missing score components in the final study cohort.

| **Variable** | **SIRS** | **qSOFA** | **NEWS** | **SOFA** | **All (N=10942)** | **Non-ICU (N=7193)** | **ICU (N=3749)** |
| --- | --- | --- | --- | --- | --- | --- | --- |
| **Temperature, n (%)** | X |  | X |  | 6 (<0.1) | 4 (<0.1) | 2 (<0.1) |
| **Respiratory rate, n (%)** | X | X | X |  | 5 (<0.1) | 4 (<0.1) | 1 (<0.1) |
| **Heart rate, n (%)** | X |  | X |  | 3 (<0.1) | 3 (<0.1) | 0 (<0.1) |
| **SBP, n (%)** |  | X | X |  | 5 (<0.1) | 3 (<0.1) | 2 (<0.1) |
| **MAP, n (%)** |  |  |  | X | 5 (<0.1) | 3 (<0.1) | 2 (<0.1) |
| **O2 saturation, n (%)** |  |  | X |  | 5 (<0.1) | 5 (<0.1) | 0 (<0.1) |
| **O2 supplement, n (%)** |  |  | X |  | 20 (0.2) | 19 (0.2) | 1 (<0.1) |
| **PaO2/FiO2, n (%)** |  |  |  | X | 8,489 (77) | 6,996 (97) | 1,493 (40) |
| **GCS, n (%)** |  | X | X | X | 101 (0.9) | 89 (1.2) | 12 (0.3) |
| **WBC, n (%)** | X |  |  |  | 31 (0.2) | 30 (0.4) | 1 (<0.1) |
| **Creatinine, n (%)** |  |  |  | X | 52 (0.5) | 47 (0.6) | 5 (0.1) |
| **Bilirubin, n (%)** |  |  |  | X | 3,927 (36) | 2,937 (40) | 990 (26) |
| **Platelets, n (%)** |  |  |  | X | 38 (0.4) | 35 (0.5) | 3 (0.1) |

Abbreviations: FiO2, fraction of inspired oxygen; GCS, Glasgow coma scale; ICU, intensive care unit; MAP, mean arterial pressure; NEWS, national early warning score; O2, oxygen; PaO2, partial pressure of arterial oxygen; qSOFA, quick sequential organ failure assessment; SBP, systolic blood pressure; SIRS, systemic inflammatory response syndrome; SOFA, sequential organ failure assessment; WBC, white blood cell count.
