# Supplementary material for: Comparative prognostic accuracy of sepsis scores for hospital mortality in adults with suspected infection in non-ICU and ICU at an academic public hospital
Source: PLoS One. 2019 Sep 16;14(9):e0222563. doi: 10.1371/journal.pone.0222563 (PMC6746500; doi:10.1371/journal.pone.0222563)
Supplement: S2 Table — Abbreviations: AUROC, area under the operator receiver curve; CI, confidence interval; ICU, intensive care unit; NEWS, national early warning score; qSOFA, quick sequential organ failure assessment; SIRS, systemic inflammatory response syndrome; SOFA, sequential organ failure assessment. N values correspond to the number of patients included in the analysis who were eligible to experience the outcome. (DOCX) [file pone.0222563.s002.docx]

S2 Table. Crude AUROCs and comparisons for prediction of hospital mortality outcomes.

| **Outcome** | **SIRS** | **qSOFA** | **NEWS** | **SOFA** |
| --- | --- | --- | --- | --- |
| **Mortality (N=10942)**  AUROC (95% CI) | 0.74 (0.72-0.76) | 0.81 (0.80-0.82) | 0.84 (0.83-0.85) | 0.88 (0.87-0.90) |
| *vs. SIRS* |  | <0.001 | <0.001 | <0.001 |
| *vs. qSOFA* | <0.001 |  | <0.001 | <0.001 |
| *vs. NEWS* | <0.001 | <0.001 |  | <0.001 |
| *vs. SOFA* | <0.001 | <0.001 | <0.001 |  |
| **ICU Mortality (n=3749)**  AUROC (95% CI) | 0.62 (0.60-0.64) | 0.63 (0.61-0.65) | 0.67 (0.65-0.70) | 0.80 (0.78-0.82) |
| *vs. SIRS* |  | 0.35 | <0.001 | <0.001 |
| *vs. qSOFA* | 0.35 |  | <0.001 | <0.001 |
| *vs. NEWS* | <0.001 | <0.001 |  | <0.001 |
| *vs. SOFA* | <0.001 | <0.001 | <0.001 |  |
| **Non-ICU Mortality (n=7193)**  AUROC (95% CI) | 0.67 (0.62-0.72) | 0.75 (0.71-0.79) | 0.77 (0.73-0.81) | 0.80 (0.75-0.84) |
| *vs. SIRS* |  | 0.002 | <0.001 | <0.001 |
| *vs. qSOFA* | 0.002 |  | 0.07 | 0.07 |
| *vs. NEWS* | <0.001 | 0.07 |  | 0.38 |
| *vs. SOFA* | <0.001 | 0.07 | 0.38 |  |

Abbreviations: AUROC, area under the operator receiver curve; CI, confidence interval; ICU, intensive care unit; NEWS, national early warning score; qSOFA, quick sequential organ failure assessment; SIRS, systemic inflammatory response syndrome; SOFA, sequential organ failure assessment. N values correspond to the number of patients included in the analysis who were eligible to experience the outcome.
