# Supplementary material for: Comparative prognostic accuracy of sepsis scores for hospital mortality in adults with suspected infection in non-ICU and ICU at an academic public hospital
Source: PLoS One. 2019 Sep 16;14(9):e0222563. doi: 10.1371/journal.pone.0222563 (PMC6746500; doi:10.1371/journal.pone.0222563)
Supplement: S3 Table — Abbreviations: AUROC, area under the operator receiver curve; CI, confidence interval; ICU, intensive care unit; NEWS, national early warning score; qSOFA, quick sequential organ failure assessment; SIRS, systemic inflammatory response syndrome; SOFA, sequential organ failure assessment. N values correspond to the number of patients included in the analysis who were eligible to experience the outcome. For ICU transfer, the reported n of 7,287 indicates the 7193 non-ICU patients and an additional 94 patients who were in the ICU at time of inclusion but had been transferred to the ICU within the preceding 24 hours and met the definition for ICU transfer. (DOCX) [file pone.0222563.s003.docx]

S3 Table. Crude AUROCs and comparisons for prediction of ICU transfer and length of stay outcomes.

| **Outcome** | **SIRS** | **qSOFA** | **NEWS** | **SOFA** |
| --- | --- | --- | --- | --- |
| **ICU Transfer (n=7287)**  AUROC (95% CI) | 0.77 (0.75-0.79) | 0.89 (0.87-0.90) | 0.94 (0.93-0.95) | 0.84 (0.81-0.86) |
| *vs. SIRS* |  | <0.001 | <0.001 | <0.001 |
| *vs. qSOFA* | <0.001 |  | <0.001 | <0.001 |
| *vs. NEWS* | <0.001 | <0.001 |  | <0.001 |
| *vs. SOFA* | <0.001 | <0.001 | <0.001 |  |
| **ICU LOS >3 days (N=10942)**  AUROC (95% CI) | 0.73 (0.72-0.74) | 0.83 (0.82-0.84) | 0.85 (0.85-0.86) | 0.84 (0.83-0.85) |
| *vs. SIRS* |  | <0.001 | <0.001 | <0.001 |
| *vs. qSOFA* | <0.001 |  | <0.001 | 0.05 |
| *vs. NEWS* | <0.001 | <0.001 |  | 0.001 |
| *vs. SOFA* | <0.001 | 0.05 | 0.001 |  |
| **LOS >7 days (N=10942)**  AUROC (95% CI) | 0.61 (0.60-0.62) | 0.63 (0.62-0.64) | 0.64 (0.63-0.65) | 0.63 (0.62-0.64) |
| *vs. SIRS* |  | <0.001 | <0.001 | 0.004 |
| *vs. qSOFA* | <0.001 |  | <0.001 | 0.97 |
| *vs. NEWS* | <0.001 | <0.001 |  | 0.03 |
| *vs. SOFA* | 0.004 | 0.97 | 0.03 |  |

Abbreviations: AUROC, area under the operator receiver curve; CI, confidence interval; ICU, intensive care unit; NEWS, national early warning score; qSOFA, quick sequential organ failure assessment; SIRS, systemic inflammatory response syndrome; SOFA, sequential organ failure assessment. N values correspond to the number of patients included in the analysis who were eligible to experience the outcome. For ICU transfer, the reported n of 7,287 indicates the 7193 non-ICU patients and an additional 94 patients who were in the ICU at time of inclusion but had been transferred to the ICU within the preceding 24 hours and met the definition for ICU transfer.
