# Supplementary figures and images for: Comparative prognostic accuracy of sepsis scores for hospital mortality in adults with suspected infection in non-ICU and ICU at an academic public hospital
Source: PLoS One. 2019 Sep 16;14(9):e0222563. doi: 10.1371/journal.pone.0222563 (PMC6746500; doi:10.1371/journal.pone.0222563)

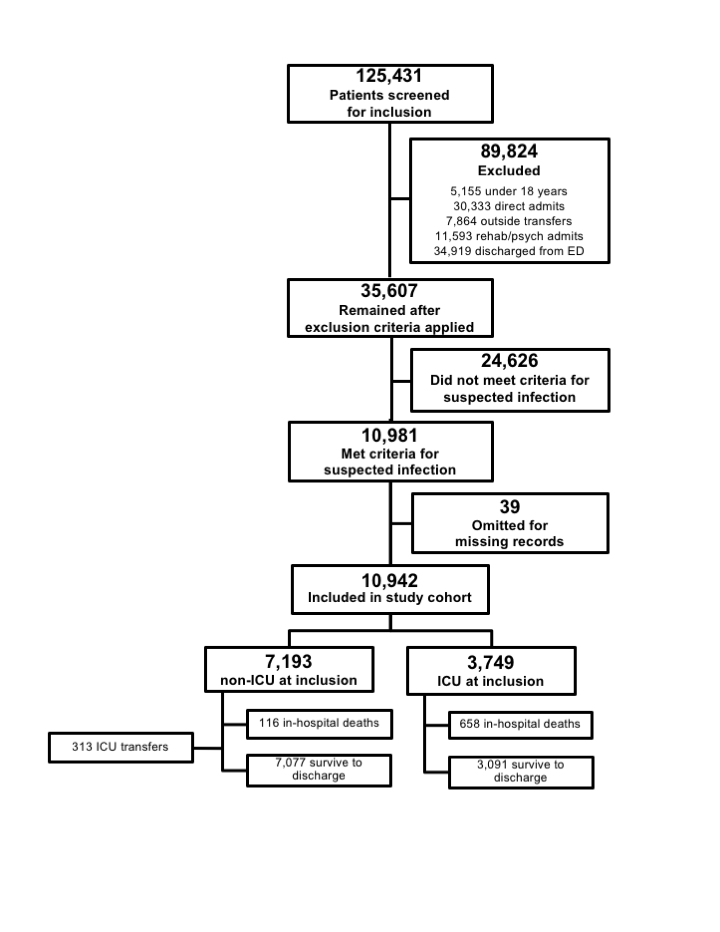

Supplement: S1 Fig — Abbreviations: ICU, intensive care unit. 125,431 patient encounters were screened for eligibility. Following exclusion of patients <18 years of age, patients who were directly admitted to the hospital or were transferred from outside institutions, were admitted to inpatient psychiatric or rehabilitation services, were evaluated in the ED and discharged, or encounters did not meet criteria for suspected infection, 10,981 patients remained. A further 39 patients (4 ICU and 35 non-ICU) were omitted from the final study cohort because all components of one of the sepsis scores were missing. The final study cohort comprised 10,942 patient encounters. (TIFF) [file pone.0222563.s004.tiff]

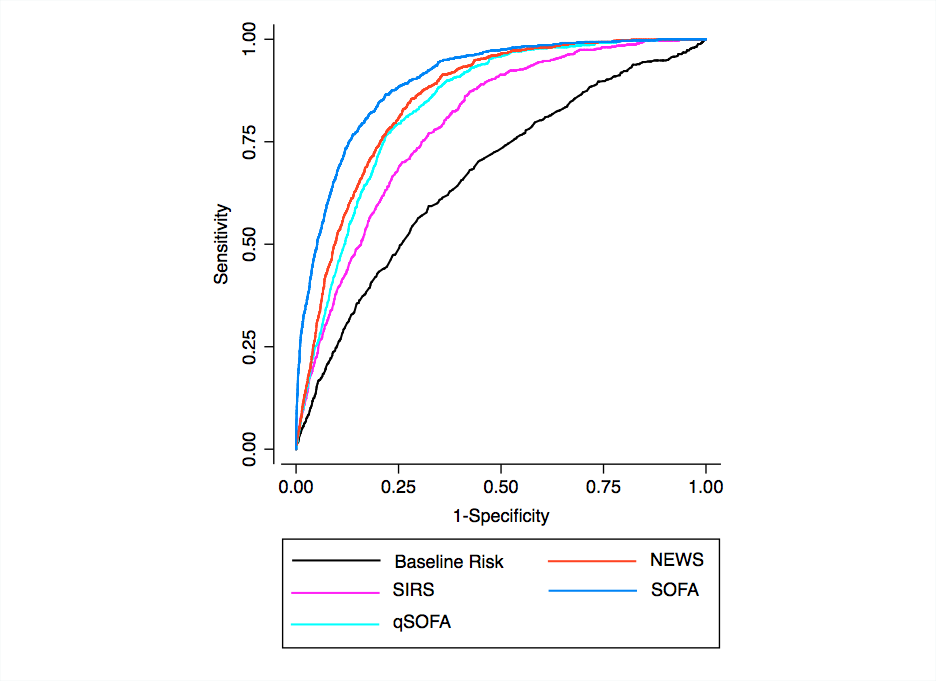

Supplement: S2 Fig — Abbreviations: NEWS, national early warning score; qSOFA, quick sequential organ function assessment; SIRS, systemic inflammatory response syndrome; SOFA, sequential organ function assessment. Adjusted AUROCs: SOFA, 0.90 (95% CI, 0.89–0.91); NEWS, 0.85 (95% CI, 0.84–0.86); qSOFA, 0.84 (95% CI, 0.83–0.85); SIRS, 0.79 (95% CI, 0.78–0.81); model of baseline risk, 0.67 (95% CI, 0.65–0.69). (TIF) [file pone.0222563.s005.tif]

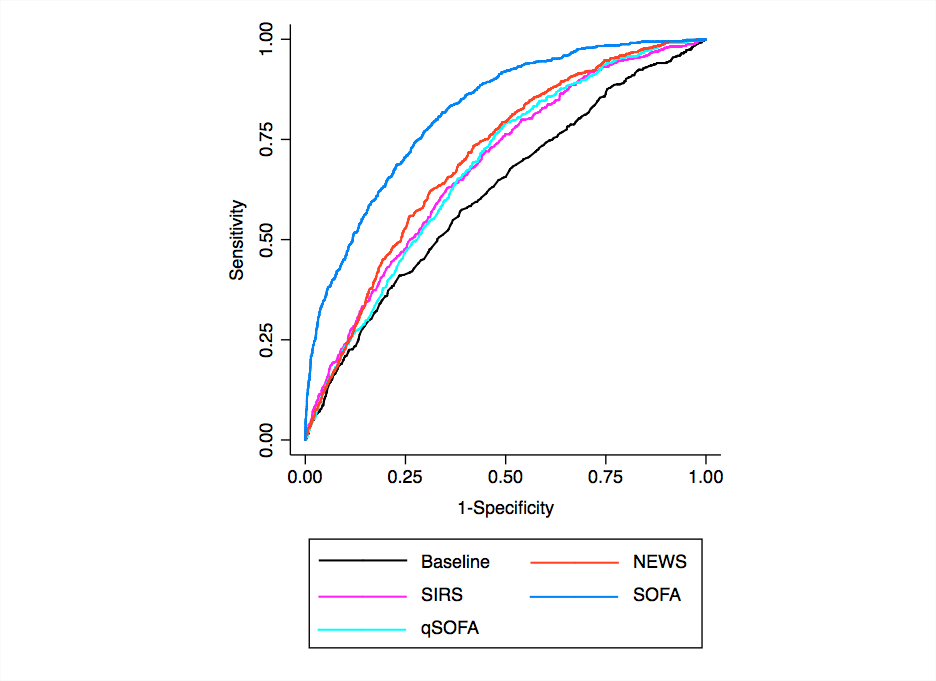

Supplement: S3 Fig — Abbreviations: NEWS, national early warning score; qSOFA, quick sequential organ function assessment; SIRS, systemic inflammatory response syndrome; SOFA, sequential organ function assessment. Adjusted AUROCs: SOFA, 0.82 (95% CI, 0.80–0.83); NEWS, 0.70 (95% CI, 0.68–0.72); qSOFA, 0.68 (95% CI, 0.66–0.70); SIRS, 0.68 (95% CI, 0.66–0.70); model of baseline risk, 0.62 (95% CI, 0.59–0.64). (TIF) [file pone.0222563.s006.tif]

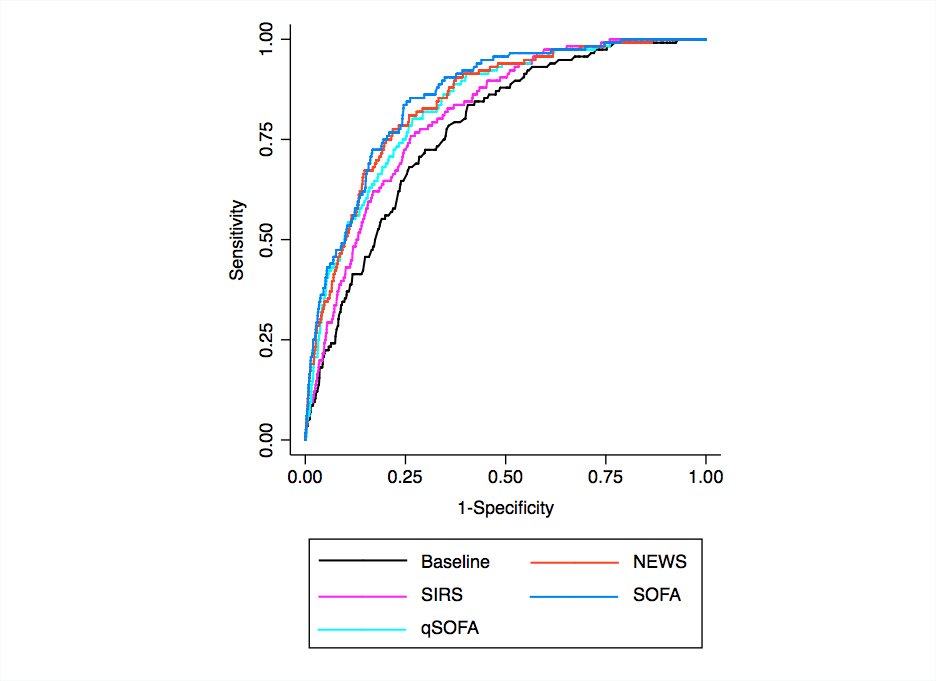

Supplement: S4 Fig — Abbreviations: NEWS, national early warning score; qSOFA, quick sequential organ function assessment; SIRS, systemic inflammatory response syndrome; SOFA, sequential organ function assessment. Adjusted AUROCs: SOFA, 0.86 (95% CI, 0.83–0.89); NEWS, 0.84 (95% CI, 0.81–0.88); qSOFA, 0.84 (95% CI, 0.80–0.87); SIRS, 0.81 (95% CI, 0.78–0.84); model of baseline risk, 0.77 (95% CI, 0.74–0.81). (TIF) [file pone.0222563.s007.tif]

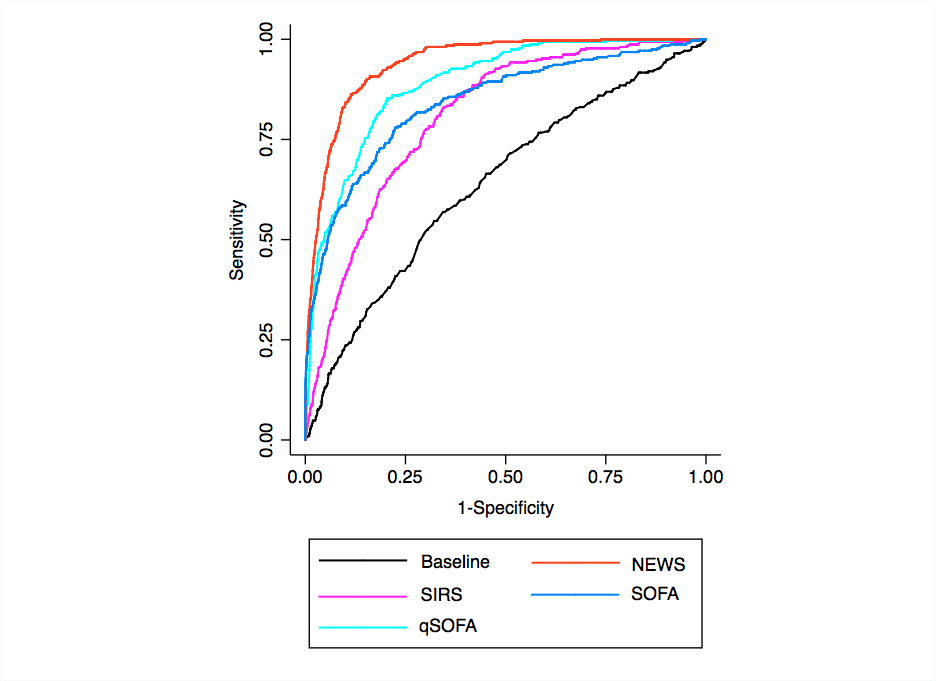

Supplement: S5 Fig — Abbreviations: NEWS, national early warning score; qSOFA, quick sequential organ function assessment; SIRS, systemic inflammatory response syndrome; SOFA, sequential organ function assessment. Adjusted AUROCs: NEWS, 0.94 (95% CI, 0.93–0.95); qSOFA, 0.89 (95% CI, 0.87–0.91); SOFA, 0.84 (95% CI, 0.82–0.87); SIRS, 0.81 (95% CI, 0.79–0.83); model of baseline risk, 0.64 (95% CI, 0.61–0.67). (TIF) [file pone.0222563.s008.tif]

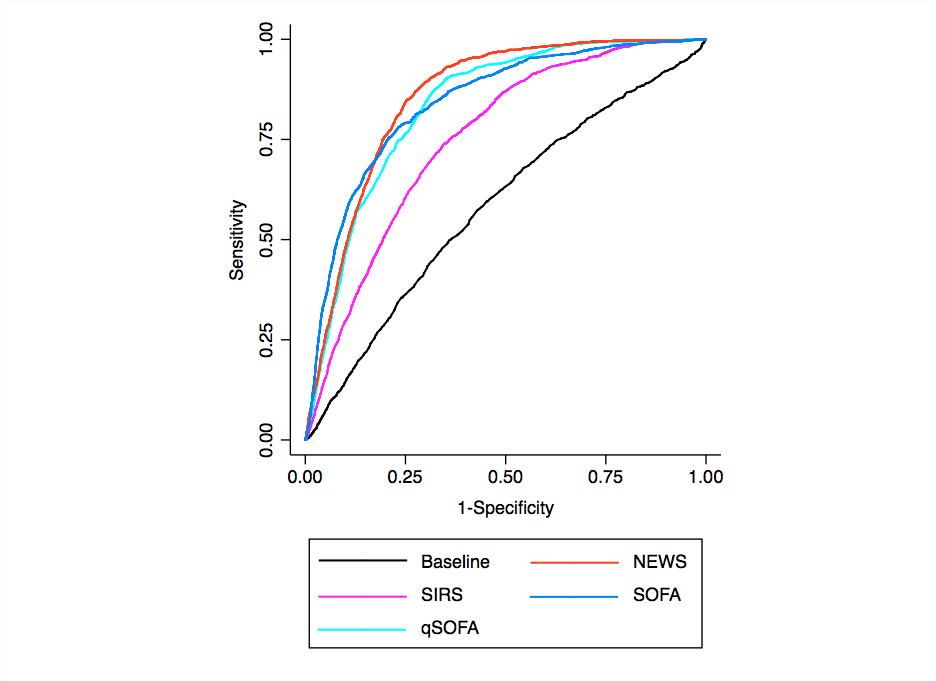

Supplement: S6 Fig — Abbreviations: NEWS, national early warning score; qSOFA, quick sequential organ function assessment; SIRS, systemic inflammatory response syndrome; SOFA, sequential organ function assessment. Adjusted AUROCs: NEWS, 0.86 (95% CI, 0.85–0.86); SOFA, 0.84 (95% CI, 0.83–0.85); qSOFA, 0.83 (95% CI, 0.83–0.84); SIRS, 0.75 (95% CI, 0.74–0.76); model of baseline risk, 0.58 (95% CI, 0.57–0.60). (TIF) [file pone.0222563.s009.tif]

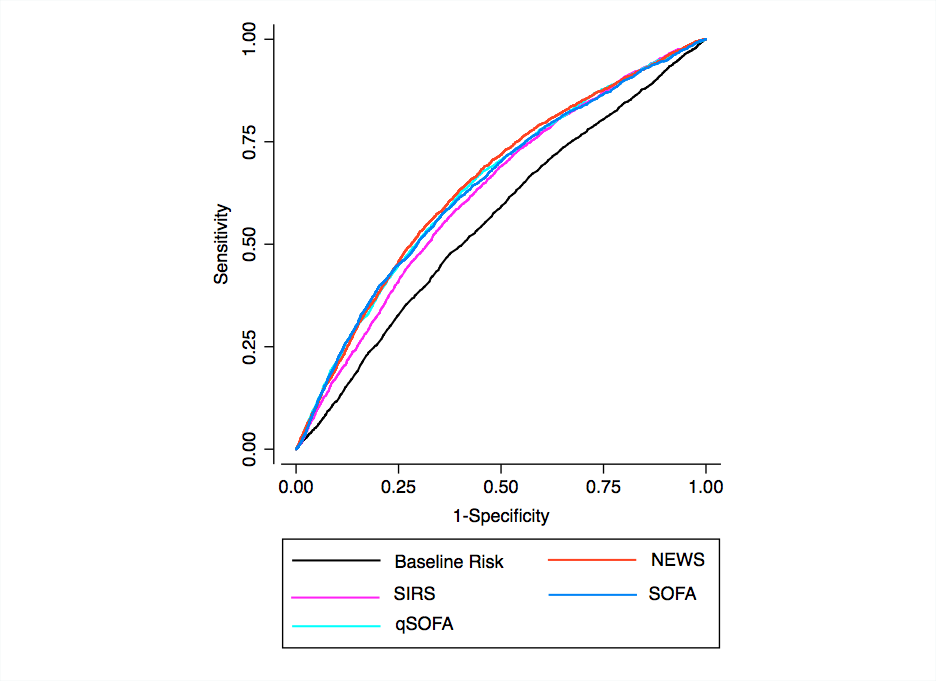

Supplement: S7 Fig — Abbreviations: NEWS, national early warning score; qSOFA, quick sequential organ function assessment; SIRS, systemic inflammatory response syndrome; SOFA, sequential organ function assessment. Adjusted AUROCs: qSOFA, 0.64 (95% CI, 0.63–0.65); NEWS, 0.65 (95% CI, 0.64–0.66); SOFA, 0.64 (95% CI, 0.63–0.65); SIRS, 0.63 (95% CI, 0.61–0.64); model of baseline risk, 0.56 (95% CI, 0.55–0.57). (TIF) [file pone.0222563.s010.tif]

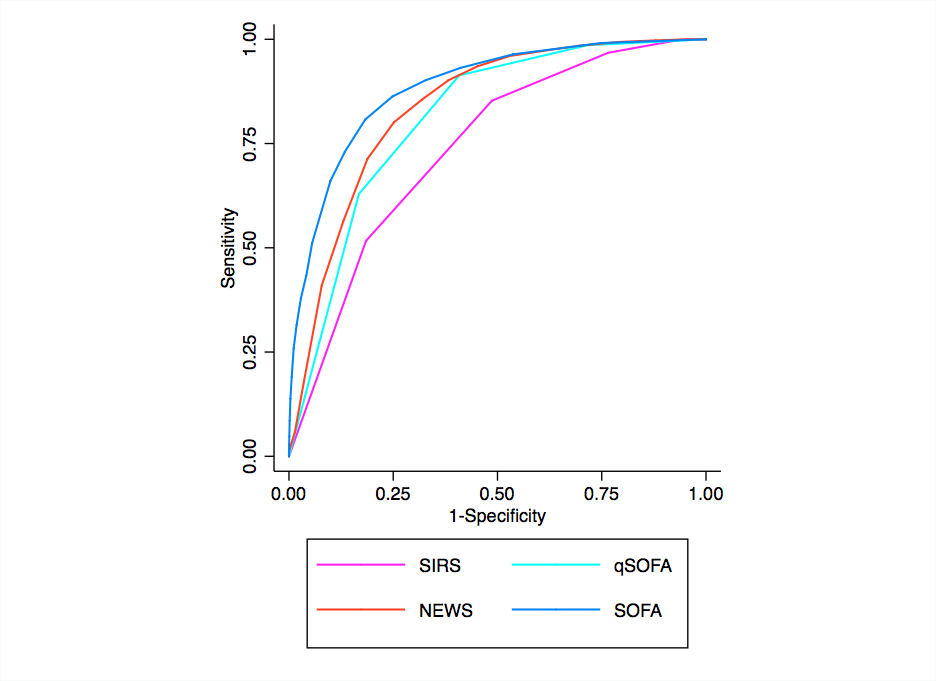

Supplement: S8 Fig — Abbreviations: NEWS, national early warning score; qSOFA, quick sequential organ function assessment; SIRS, systemic inflammatory response syndrome; SOFA, sequential organ function assessment. Crude AUROCs: SOFA 0.88 (95% CI, 0.87–0.90); NEWS, 0.84 (95% CI, 0.83–0.85); qSOFA, 0.81 (95% CI, 0.80–0.82); SIRS, 0.74 (95% CI, 0.72–0.76). (TIF) [file pone.0222563.s011.tif]

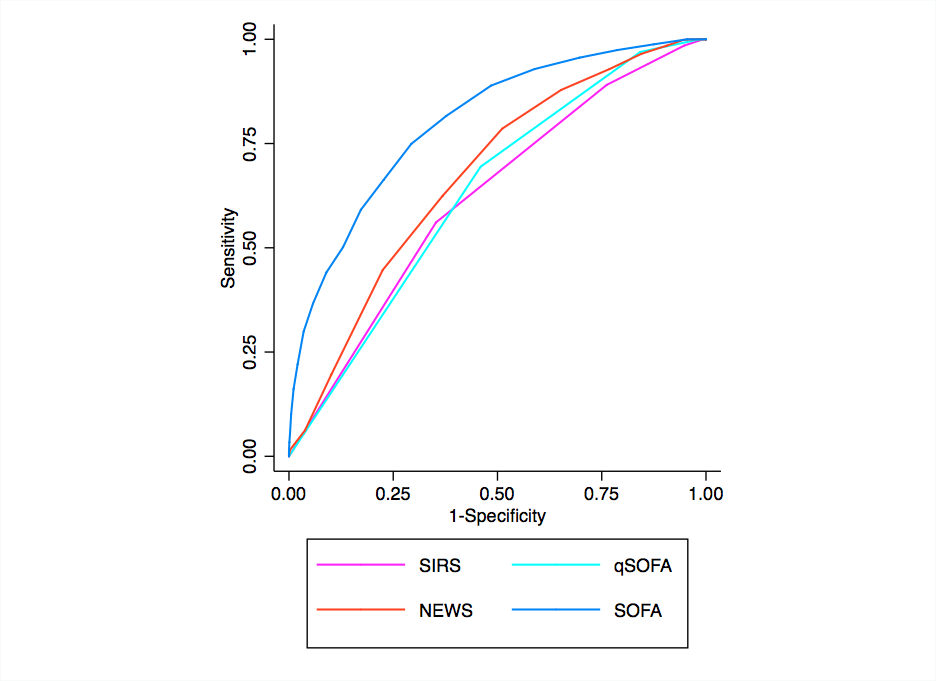

Supplement: S9 Fig — Abbreviations: NEWS, national early warning score; qSOFA, quick sequential organ function assessment; SIRS, systemic inflammatory response syndrome; SOFA, sequential organ function assessment. Crude AUROCs: SOFA, 0.80 (95% CI, 0.78–0.82); NEWS, 0.67 (95% CI, 0.65–0.70); qSOFA, 0.63 (95% CI, 0.61–0.65); SIRS, 0.62 (95% CI, 0.60–0.64). (TIF) [file pone.0222563.s012.tif]

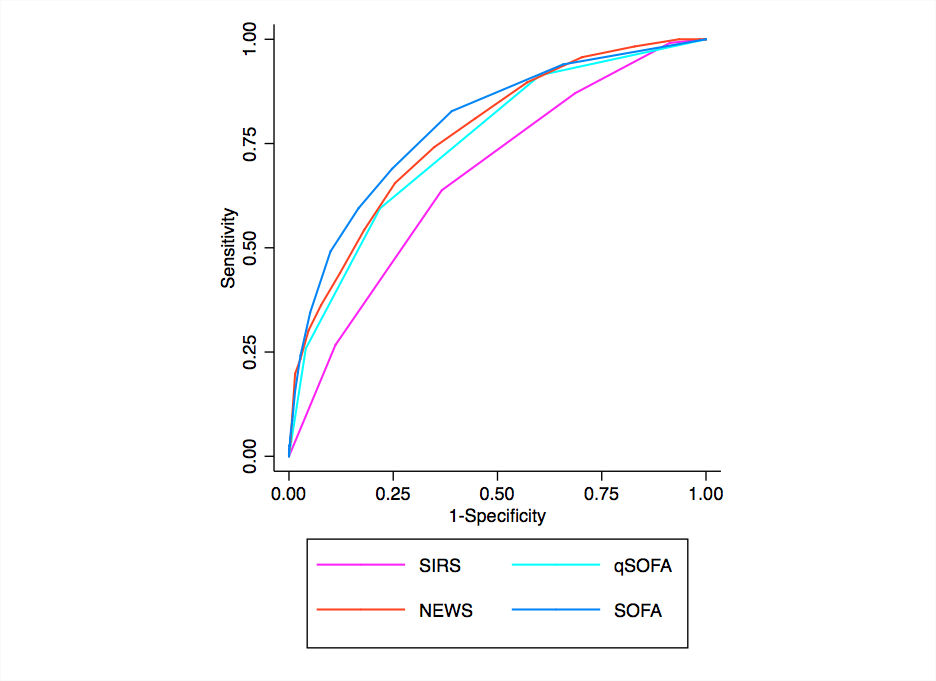

Supplement: S10 Fig — Abbreviations: NEWS, national early warning score; qSOFA, quick sequential organ function assessment; SIRS, systemic inflammatory response syndrome; SOFA, sequential organ function assessment. Crude AUROCs: SOFA, 0.80 (95% CI, 0.75–0.84); NEWS, 0.77 (95% CI, 0.73–0.81); qSOFA, 0.75 (95% CI, 0.71–0.79); SIRS, 0.67 (95% CI, 0.62–0.72). (TIF) [file pone.0222563.s013.tif]

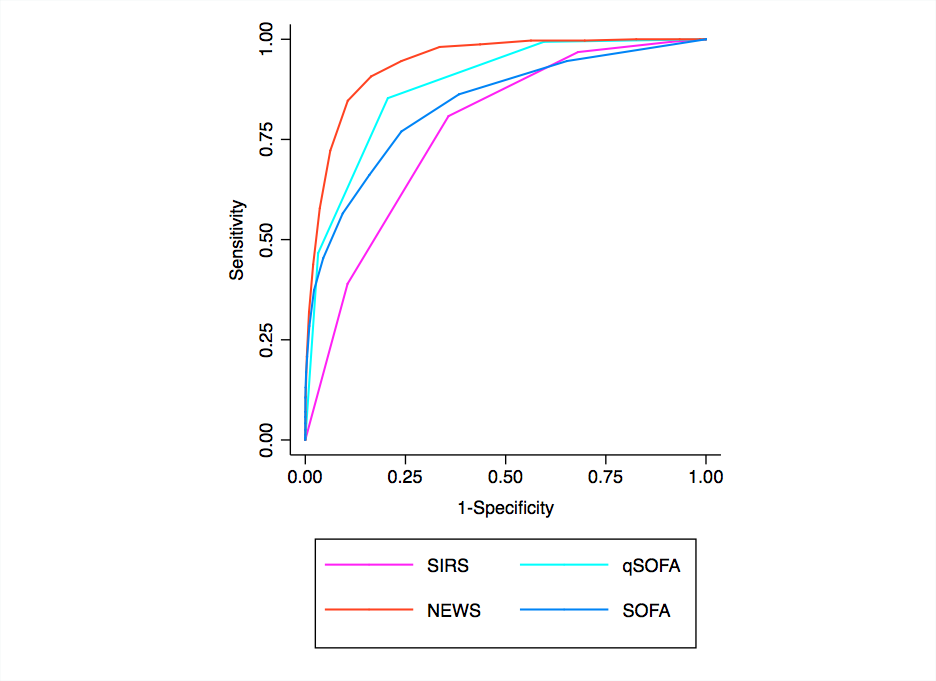

Supplement: S11 Fig — Abbreviations: NEWS, national early warning score; qSOFA, quick sequential organ function assessment; SIRS, systemic inflammatory response syndrome; SOFA, sequential organ function assessment. Crude AUROCs: NEWS, 0.94 (95% CI, 0.93–0.95); qSOFA, 0.89 (95% CI, 0.87–0.90); SOFA, 0.84 (95% CI, 0.81–0.86); SIRS, 0.77 (95% CI, 0.75–0.79). (TIF) [file pone.0222563.s014.tif]

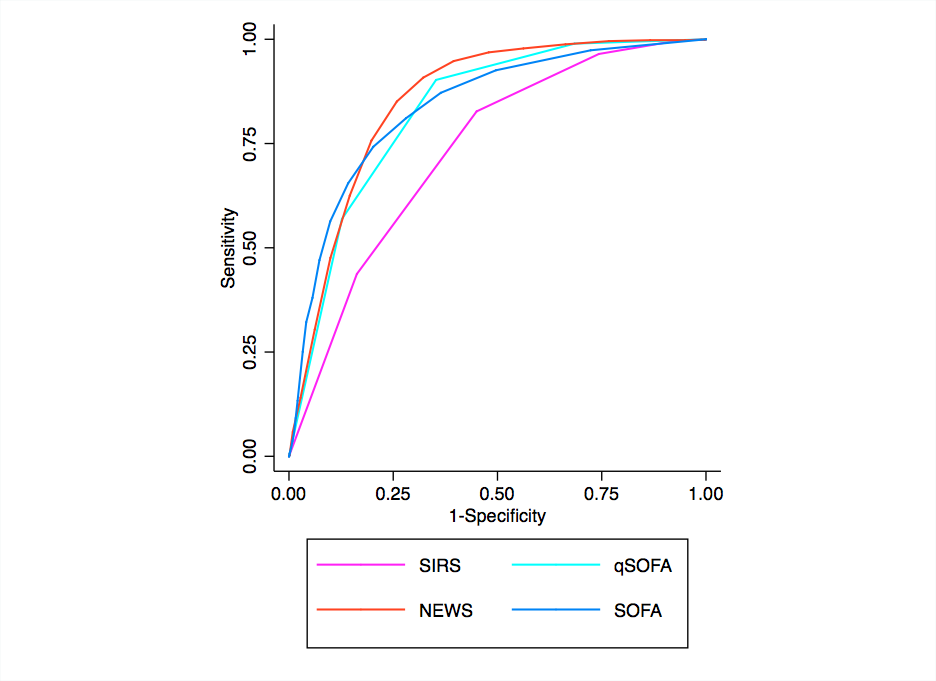

Supplement: S12 Fig — Abbreviations: NEWS, national early warning score; qSOFA, quick sequential organ function assessment; SIRS, systemic inflammatory response syndrome; SOFA, sequential organ function assessment. Crude AUROCs: NEWS, 0.85 (95% CI, 0.85–0.86); SOFA, 0.84 (95% CI, 0.83–0.85); qSOFA, 0.83 (95% CI, 0.82–0.84); SIRS, 0.73 (95% CI, 0.72–0.74). (TIF) [file pone.0222563.s015.tif]

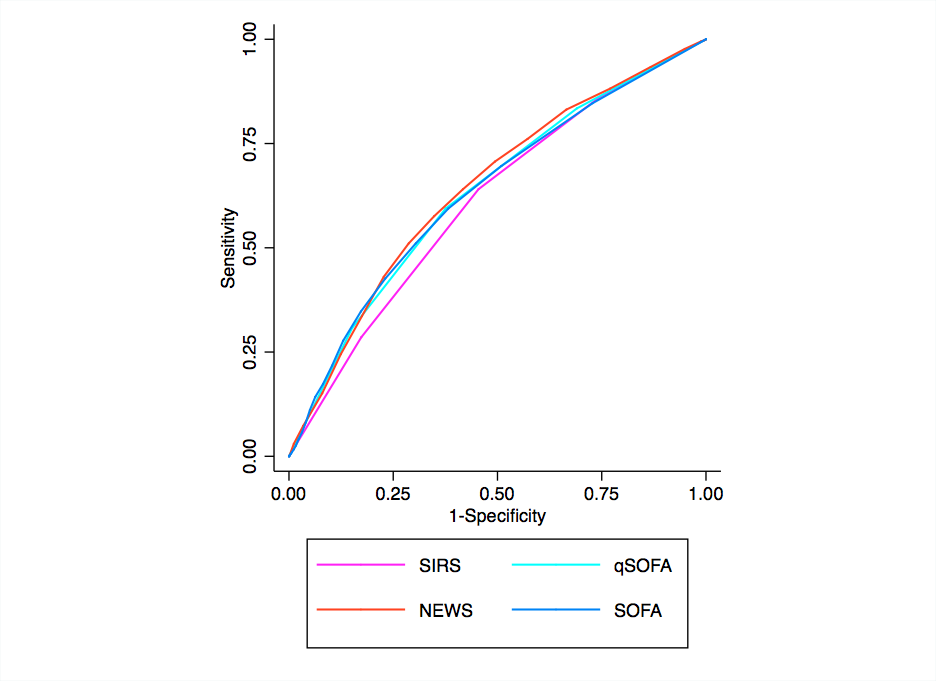

Supplement: S13 Fig — Abbreviations: NEWS, national early warning score; qSOFA, quick sequential organ function assessment; SIRS, systemic inflammatory response syndrome; SOFA, sequential organ function assessment. Crude AUROCs: qSOFA, 0.63 (95% CI, 0.62–0.64); NEWS, 0.64 (95% CI, 0.63–0.65); SOFA, 0.63 (95% CI, 0.62–0.64); SIRS, 0.61 (95% CI, 0.60–0.62). (TIF) [file pone.0222563.s016.tif]

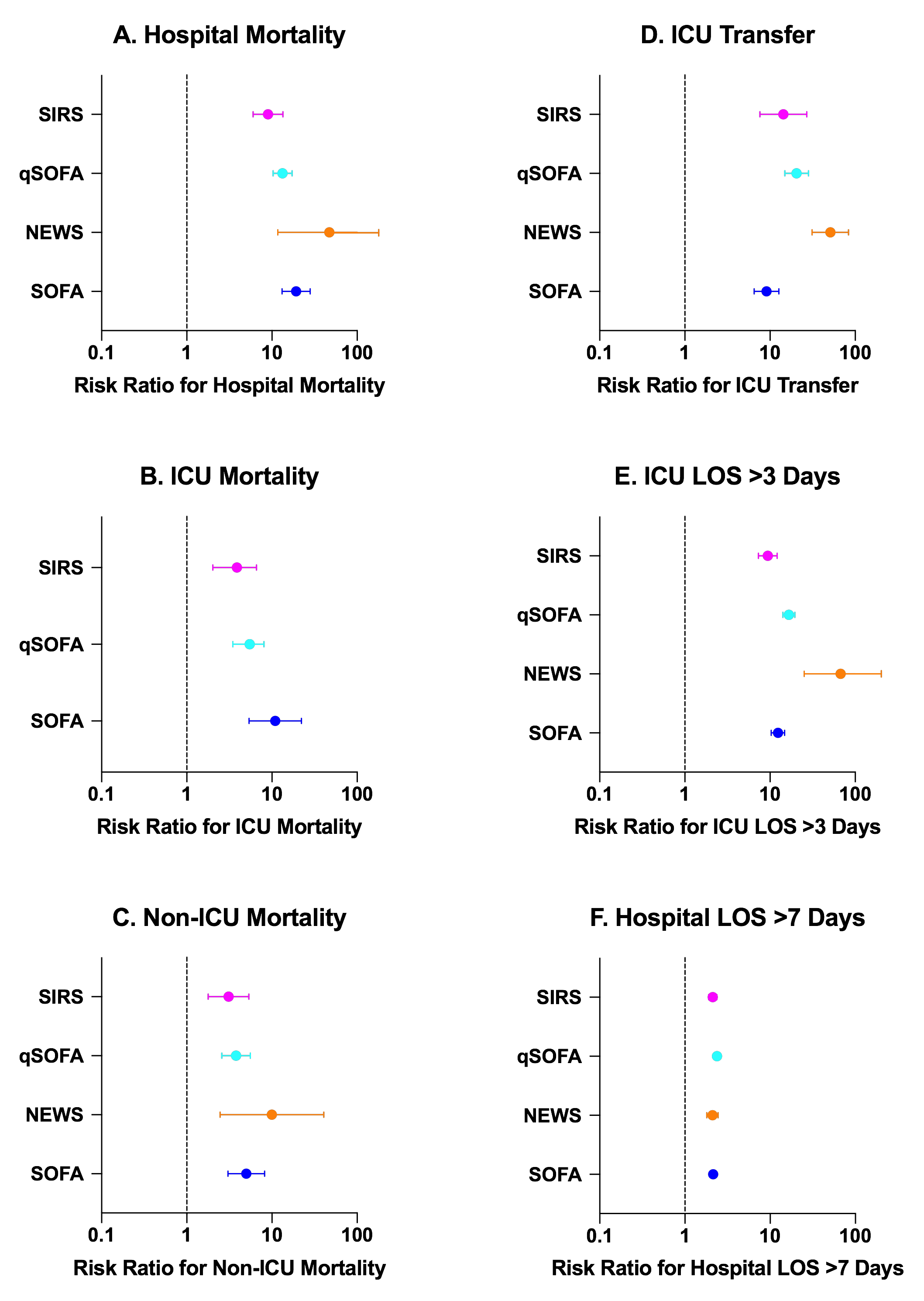

Supplement: S14 Fig — Abbreviations: ICU, intenstive care unity; LOS, length of stay; NEWS, national early warning score; qSOFA, quick sequential organ function assessment; RR, risk ratio; SIRS, systemic inflammatory response syndrome; SOFA, sequential organ function assessment. Criteria for score positivity: SIRS ≥2, qSOFA ≥2, NEWS ≥5, SOFA ≥2. A) Adjusted risk ratios for hospital mortality: NEWS, 47.0 (95% CI, 11.7–188.8), SOFA, 19.2 (95% CI, 13.1–28.1); qSOFA, 13.3 (95% CI, 10.3–17.2); SIRS, 9.0 (95% CI, 6.0–13.5). B) Adjusted risk ratios for ICU mortality: SOFA, 10.9 (95% CI, 5.4–22.2); qSOFA, 5.5 (95% CI, 3.5–8.1); SIRS, 3.9 (95% CI, 2.0–6.6). No patients with NEWS <5 died in the ICU and thus risk ratio of NEWS for ICU mortality could not be calculated; therefore NEWS is omitted from panel B. C) Adjusted risk ratios for non-ICU mortality: NEWS, 10.0 (95% CI, 2.5–40.6); SOFA, 5.0 (95% CI, 3.0–8.2); qSOFA, 3.8 (95% CI, 2.6–5.6); SIRS, 3.1 (95% CI, 1.8–5.4). D) Adjusted risk ratios for ICU transfer: NEWS, 51.0 (95% CI, 31.1–83.5); qSOFA, 20.5 (95% CI, 14.9–28.3); SIRS, 14.3 (95% CI, 7.6–27.0); SOFA, 9.1 (95% CI, 6.5–12.7). E) Adjusted risk ratios for ICU LOS >3 days: NEWS, 67.4 (95% CI, 25.2–180.1); qSOFA, 16.6 (95% CI, 14.1–19.5); SOFA, 12.4 (95% CI, 10.3–14.9); SIRS, 9.4 (95% CI, 7.3–12.1). F) Adjusted risk ratios for hospital LOS >7 days: qSOFA, 2.4 (95% CI, 2.2–2.6); SOFA, 2.1 (95% CI, 2.0–2.3); SIRS, 2.1 (95% CI, 1.9–2.4); NEWS, 2.1 (95% CI, 1.8–2.4). Error bars represent 95% confidence interval. (TIFF) [file pone.0222563.s017.tiff]
